# Neolithic cultivation of water chestnuts (*Trapa* L.) at Tianluoshan (7000-6300 cal BP), Zhejiang Province, China

**DOI:** 10.1038/s41598-017-15881-w

**Published:** 2017-11-24

**Authors:** Yi Guo, Rubi Wu, Guoping Sun, Yunfei Zheng, Benjamin T. Fuller

**Affiliations:** 1 Department of Cultural Heritage and Museology, School of Humanities, Zhejiang University, Hangzhou, 310028 China; 2 Zhejiang Provincial Research Institute of Cultural Relics and Archaeology, Hangzhou, 310014 China; 3 Department of Archaeology and Anthropology, University of Chinese Academy of Sciences, Beijing, 100049 China

## Abstract

Water chestnuts (*Trapa*) are frequently recovered at Neolithic sites along the Lower Yangtze River Valley and have been important components of the diets of prehistoric people. However, little systematic research has been conducted to determine their cultural and dietary importance. Excavations at the Tianluoshan site produced large quantities of well-preserved specimens, which provide an excellent collection for studying morphological changes with time. Using modern wild and domesticated water chestnuts (n = 447) as a reference, we find Neolithic samples (n = 481) at Tianluoshan are similar in shape but smaller in size compared to the domesticated species *Trapa bispinosa*. In particular, the Tianluoshan water chestnuts have bigger seeds than the wild species *Trapa incisa*. Further, water chestnuts diachronically increased in size at the Tianluoshan site with significant differences (one-way, ANOVA) observed for length (p = 7.85E-08), height (p = 3.19E-06), thickness (p = 1.2E-13), top diameter (p = 5.04E-08) and bottom diameter (p = 1.75E-05) between layers 7 (6700-6500 cal BP) and 6 (6500–6300 cal BP). These results suggest that water chestnuts were actively selected based on size (big), shape (full fruit, two round horns, wide base, etc.) and were an important non-cereal crop to the agricultural practices at the Tianluoshan site.

## Introduction

The water chestnut (*Trapa* L.) is an edible plant that grows widely in Eurasia, Africa, North America and Australia^1,2^. In China, this plant is mostly distributed in the subtropical region of the Yangtze River drainage basin^3–5^. Water chestnuts have a long history of utilization^6,7^, and the early use of water chestnuts for human consumption is found at many locations across China. Currently, there are 21 Chinese Neolithic sites where water chestnuts have been discovered (Table 1). This indicates that water chestnuts were one of an important number of foods for the ancient people of China, especially for the inhabitants of the Middle and Lower Yangtze River Valleys^8^. In addition, rice remains are normally found associated with the water chestnuts^8^, suggesting that the production and utilization of water chestnuts share common characteristics with rice agricultural systems.

**Table 1:** Summary of Neolithic sites in China that have found prehistoric water chestnuts remains.

**No**.	Site	Species	Archaeological Culture	Age (cal BP)	City, Province	Comments	References
1	Jiahu	?	Jiahu	9000-7800	Wuyang, Henan	more than 7000 pieces	15,16
2	Bashidang	?	Pengtoushan	8000-7500★	Lixian, Hunan	150 half-fruit	53,54
3	Chengtoushan	*Trapa maximowiezii* Korsh.	Pengtoushan	6500-4800★	Lixian, Hunan		13
4	Kuahuqiao	*T. bicornis* Osbeck var. *bicornis, T. quadrispinosa* Roxb.	Kuahuqiao	8000-7000	Xiaoshan, Zhejiang		41,42
5	Xiasun	?	Kuahuqiao	8000-7000	Xiaoshan, Zhejiang		41,42
6	Hemudu	*Trapa bispinosa* Roxb.	Hemudu	7000-5800	Yuyao, Zhejiang	shells	55
7	Tianluoshan	*Trapa bispinosa* Roxb.	Hemudu	7000-5800	Yuyao, Zhejiang		18
8	Fujiashan	?	Hemudu	7000-5800	Ningbo, Zhejiang		17
9	Majiabang	*Trapa acornis* Nakano	Majiabang	7000-5800	Jiaxing, Zhejiang		8
10	Luojiajiao	?	Majiabang	7000-5800	Tongxiang, Zhejiang		11
11	Xinqiao	*Trapa maximowiezii* Korsh.	Majiabang	7000-5800★	Tongxiang, Zhejiang	13 horns, average length 13 mm, carbonized	56
12	Qiucheng	*Trapa acornis* Nakano	Majiabang	7000-5800★	Wuxing, Zhejiang		8
13	Caoxieshan	*Trapa acornis* Nakano	Majiabang	7000-5800	Wuxian, Jiangsu	stems and fruits	57
14	Longqiuzhuang	?	Longqiuzhuang	7000-5500	Gaoyou, Jiangsu		58–60
15	Yuhuazhai	?	Banpo and Shijia Periods	6500-5500	Xi’an, Shaanxi		14
16	Chuodun	?	Majiabang to Maqiao	6300-3300	Kunshan, Jiangsu		13
17	Longnan	?	Songze to Liangzhu	5360-4760	Wujiang, Jiangsu		61
18	Bianjiashan	?	Liangzhu	5300-4200	Yuhang, Zhejiang		62
19	Qingdun	?	Liangzhu	5300-4200	Hai’an, Jiangsu		8,63
20	Qianshanyang	*T. acornis* Nakano, *T. maximowiezii* Korsh., *T. quadrispinosa* Roxb.	Qianshanyang	4400-4200	Wuxing, Zhejiang		65,66
21	Guangfulin	*Trapa bispinosa* Roxb.	Guangfulin	4200-4000	Shanghai	charred fruits, flesh and pieces of shells; 360 fragments	66,67

Note: ★ = uncalibrated age as original dating information was not available.

Due to a lack of systematic research on Chinese water chestnuts, there are many questions associated with the timing, places and processes by which water chestnuts were domesticated as well as its importance in the diets of prehistoric people^8–12^. In the past, many scholars have identified water chestnuts as a wild food that was gathered, rather than actively selected for cultivation^13,14^. This was certainly the case for many Neolithic sites. For example, the more than 7000 pieces of water chestnuts unearthed at the Jiahu site (9000-7800 cal BP, Jiahu Culture) are associated with the wild variety and are believed to have played an important role in the gathering economy of these people^15,16^. However, at the Qiucheng site (7000-5500 BP, Majiabang Culture) water chestnuts are similar in shape but smaller in size to the modern domesticated Nanhu water chestnuts which are the primary variety cultivated in Jiaxing City^8^. In addition, water chestnuts from the Fujiashan site (6500–4500 cal BP, Hemudu Culture) are regarded as domesticated water chestnuts^17^.

**Figure 1:**
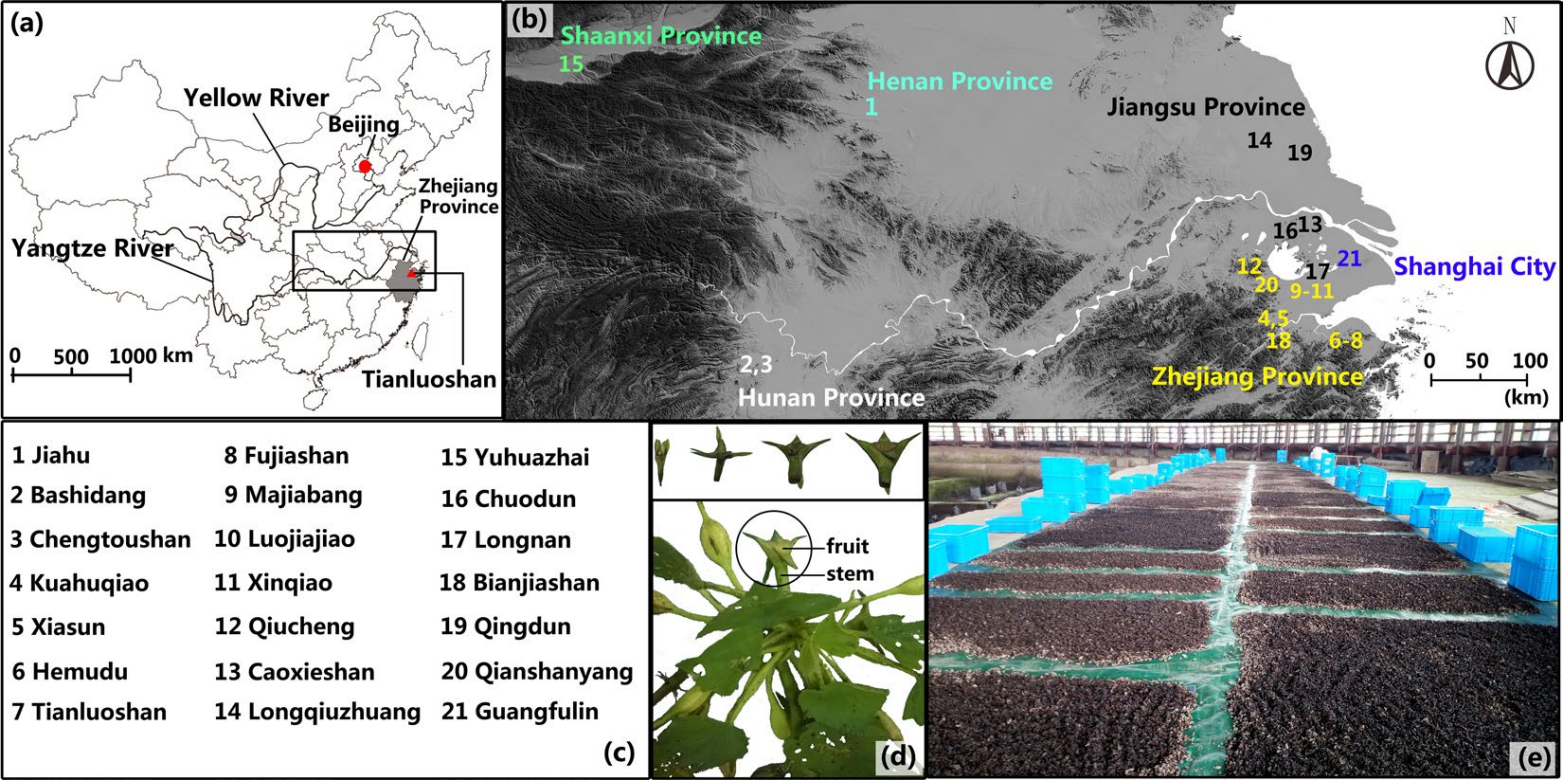
(**a**) Map of the Tianluoshan site in China. (**b**) Map and (**c**) list of Chinese Neolithic sites with water chestnuts. (All maps created by the author Rubi Wu, using Global Mapper and Adobe Photoshop version: 12.0.1, http://www.adobe.com/cn/products/photoshop.html?promoid=1NZGDDSP&mv=other&origref=http%3A%2F%2F). (**d**) Photo of modern wild water chestnut fruit on the stem and its basic growth process, inset. (**e**) Photo of water chestnut remains at the Tianluoshan site.

The Tianluoshan site (7000–5800 cal BP) is located in Yuyao City, Zhejiang Province, China, and is about 120 km southeast from the modern city of Hangzhou (Fig. 1a). It was an important site of the Hemudu Culture, where plant, animal and human remains were recovered. Since 2004, eight cultural levels were excavated, and in the lower part of the site below layer 6, the artifacts are sealed in a waterlogged and anaerobic environment that results in exceptional preservation of organic material^18^. Abundant plant remains have been recovered such as: rice *(Oryza rufipogon/sativa*), water chestnut (*Trapa bispinosa*), acorn (*Quercus* spp. (*sensu lato*)), foxnut (*Euryale ferox*), peach (*Prunus persica*), bottle gourd (*Lagenaria siceraria*), etc^19^. Many of the water chestnuts found during the excavation are fragments that represent refuse from human consumption with a comparatively small number of intact specimens. However, in pits where the water chestnuts were stored, they were found completely intact. This large number of waterlogged and well-preserved water chestnuts recovered from the different cultural layers at Tianluoshan (Fig. 1e) permits the investigation of how these plants may have been selected for advantageous traits for human consumption through time.

While past archaeobotanical studies have examined the use of wild and domesticated plant resources in many regions such as the Near East^20–22^ and Central Europe^23–25^ (where the preservation of specimens can also be waterlogged), there has been less research attention focused on non-grain crop domestication processes in China. In order to address this topic in more detail, we measure the morphological characteristics of water chestnuts at the Tianluoshan site and compare these results with modern wild and domestic varieties from eastern China. The goal of this research is to investigate possible diachronic changes in the size, shape and type (wild or domestic) of the water chestnuts. By comparing the Neolithic and modern water chestnuts, we can examine the process of water chestnut cultivation at Tianluoshan, which will provide new information about how non-cereal crops were influenced by humans in China.

## Results

All sample information and measurements are listed and summarized in the Supplementary Tables 1 and 2. Comparison of the waterlogged and preserved samples (see Methods section below) from pit H69 (layer 6) found no statistical differences for the five measured parameters except for the top diameter (p = 0.017, one-way ANOVA), which was larger in the preserved specimens (Table 2). This suggests that caution should be applied in the interpretation of this parameter. However, the effects of preservation were not significantly different for the other measurements and the two groups were treated equally. The water chestnut measurements are grouped according to archaeological layer in Table 3, and are found to increase in size from layers 8 to 6. No statistical differences were found between the measurements of layers 8 and 7. However, significant differences (one-way, ANOVA) were observed for length (p = 7.85E-08), height (p = 3.19E-06), thickness (p = 1.2E-13), top diameter (p = 5.04E-08) and bottom diameter (p = 1.75E-05) between layers 7 and 6.

**Table 2:** Results of one-way ANOVA on Neolithic water chestnuts conserved in two ways (unit: mm)

Pits	Time (cal BP)	n	Length	n	Height	n	Thickness	n	TopDiameter	n	Bottom Diameter
H69⑥ Waterlogged	6500-6300	262	32.21 ± 3.46^a^	262	16.90 ± 2.17^a^	259	13.60 ± 2.70^a^	262	6.48 ± 0.91^a^	246	11.17 ± 2.57^a^
H69⑥ Preserved	6500-6300	52	32.65 ± 3.44^a^	53	17.48 ± 2.34^a^	50	13.74 ± 2.73^a^	53	6.83 ± 1.20^b^	51	11.73 ± 2.80^a^

Note: a,b are used to show the outcome of the one-way ANOVA, the same letter under the same column means no significant difference.

**Table 3:** Results of one-way ANOVA on Neolithic water chestnuts summed by layers (unit: mm). Estimated ages of the layers are based on radiocarbon dates from Wu *et al*.^68^ and Jin *et al*.^52,69^.

Layer	Time (cal BP)	n	Length	n	Height	n	Thickness	n	Top Diameter	n	Bottom Diameter
8	7000-6700	46	28.65 ± 3.92^a^	45	15.30 ± 2.29^a^	37	11.71 ± 2.53^a^	46	5.64 ± 0.70^a^	42	9.31 ± 2.92^a^
7	6700-6500	123	30.10 ± 4.32^b^	125	15.84 ± 2.62^a^	123	11.29 ± 3.21^a^	125	5.94 ± 1.11^a^	124	9.99 ± 3.03^a^
6	6500-6300	314	32.28 ± 3.50^c^	315	17.00 ± 2.21^b^	309	13.62 ± 2.70^b^	315	6.54 ± 0.97^b^	297	11.26 ± 2.62^b^

Note: a,b,c are used to show the outcome of the one-way ANOVA, the same letter under the same column means no significant difference.

**Figure 2:**
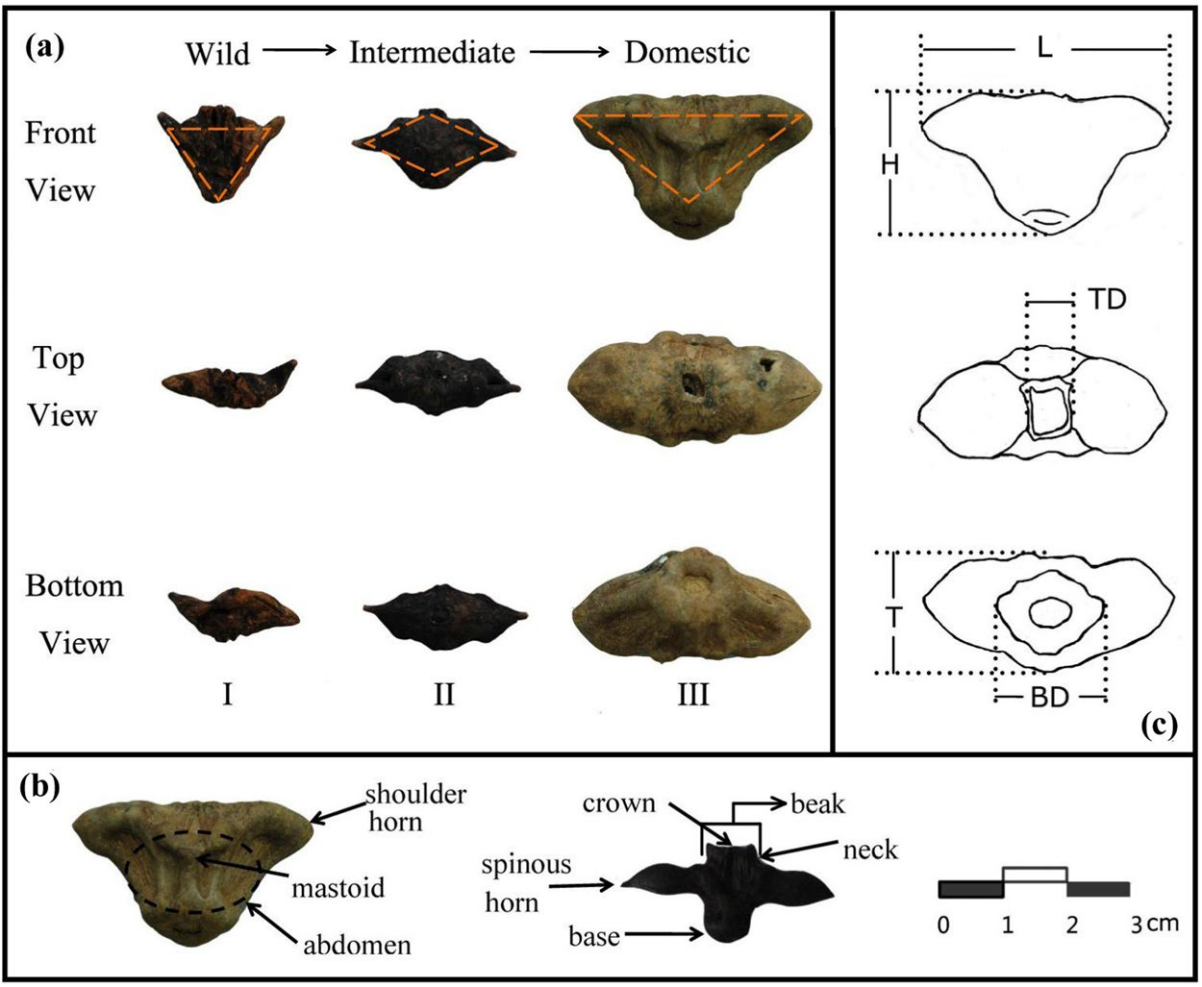
(**a**) Photographs that represent the three general shapes of classification for Type I (wild), Type II (intermediate) and Type III (domestic) water chestnuts. (**b**) Photographs depicting the specific regions of the water chestnut based on the work of Wang *et al*.^49^. (**c**) Diagram showing the measurement locations of the water chestnuts. L: length; H: height; TD: top diameter; T: thickness; BD: bottom diameter.

In terms of shape characteristics (Table 4) (classification methods can be found in the Method section), the majority of the Tianluoshan water chestnuts (~77%) belong to Type III. They have a relatively large size in comparison to the other types, and have the shape of an inverted triangle (Fig. 2a). The fruits are plump and the shoulder horns are short, round and not spinous. The beak is not distinct and the base connected to the stem is wide. The Type II specimens (~20%) have the shape of a diamond and possess a big crown and neck. In addition, few Type II specimens possess mastoids on their abdomen. The two shoulder horns point horizontally or at a slight angle upwards and have short, sharp and hard tips (usually together with big horns). The Type I water chestnuts or the wild variety are found in the smallest numbers (~4%) and are generally smaller in size with a narrow-inverted-triangle shape and a crown on the top. The shoulder horns slant upward and have hard tips while the base is generally shorter than the Type II samples. Most of the Type I specimens have mastoids on their abdomen, and bases that are similar to those of Type II.

**Table 4:** Distribution of the shapes of the Neolithic water chestnuts at Tianluoshan.

Location	Type I (Wild) N (%)	Type II (Intermediate) N (%)	Type III (Domesticated) N (%)	Sum of Samples
T104 Layer 8 Preserved	2 (28.6%)	5 (71.4%)	0 (0%)	7
T206 Layer 8 Preserved	3 (7.9%)	4 (10.5%)	31 (81.6%)	38
T205 Layer 7 Waterlogged	4 (5.5%)	12 (16.4%)	57 (78.1%)	73
T305 Layer 7 Waterlogged	3 (6%)	11 (22%)	36 (72%)	50
H69 Layer 6 Waterlogged	3 (1.1%)	54 (20.7%)	204 (78.1%)	261
H69 Layer 6 Preserved	2 (3.8%)	7 (13.5%)	43 (82.7%)	52
Total	**17 (3.5%)**	**93 (19.3%)**	**371 (77.1%)**	**481**

## Discussion

Water chestnuts have a long and stable presence in the archaeobotanical assemblage at Tianluoshan^13,26^. The large quantities of intact water chestnuts from storage pits, as well as the broken pieces from human consumption, reflect the important role that this plant played in the human diet.

**Figure 3:**
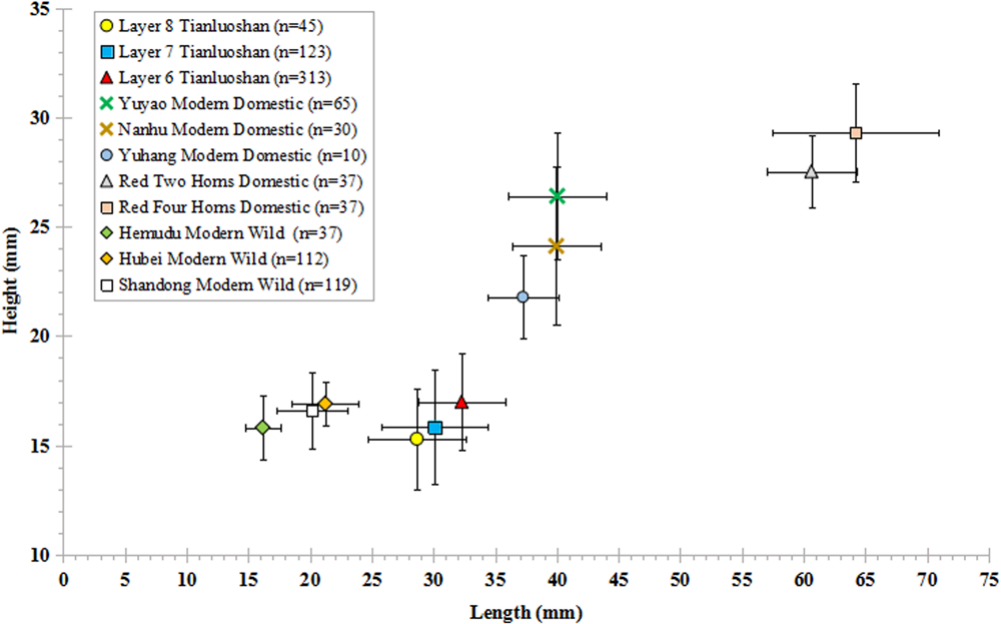
The relationship between length and height of Neolithic Tianluoshan and modern water chestnuts from China.

**Figure 4:**
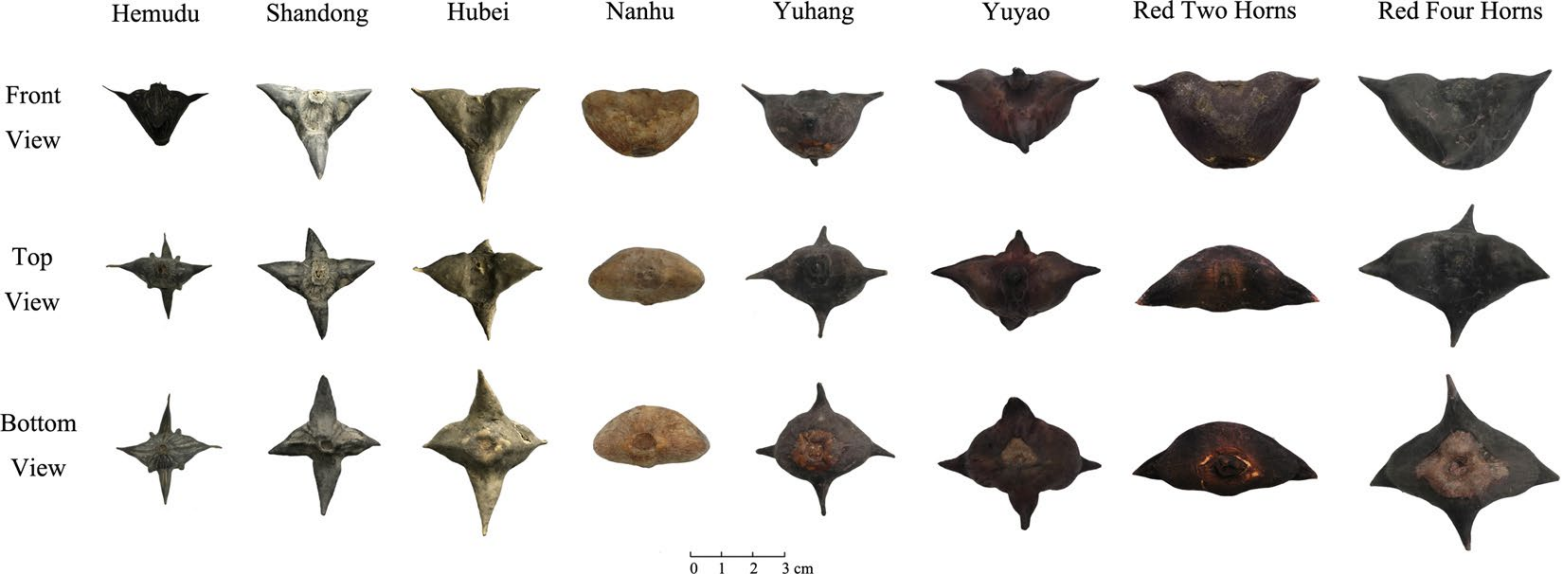
Photograph of the modern water chestnuts measured in this study.

Diachronic increases in the size of the Tianluoshan water chestnuts from layers 8 to 6 are shown in Fig. 3. These archaeological specimens are smaller than the modern domestic types but remarkably bigger than the modern wild types. In terms of shape, Type III (full fruit, two round horns, wide base, etc.) occupied the dominant position at Tianluoshan for at least 700 years. Comparing the Neolithic water chestnuts with the modern Chinese varieties, specimens at Tianluoshan are relatively similar in shape with the domesticated species *Trapa bispinosa* Roxb., but distinctly different from the wild species, *Trapa incisa* Sieb. and Zucc. (Fig. 4).

According to modern botanical research genetic polymorphisms are found in water chestnuts, and they are capable of generating new variants in the wild^27,28^. In the absence of human intervention, they will not consistently produce the same size and shape of fruit if subjected to variable and/or unfavorable growing conditions^29–31^. For example, in order to maintain the desirable traits of the Nanhu water chestnuts (a highly domesticated species without sharp horns), modern farmers need to accomplish a series of activities at specific growing times such as: picking the water chestnuts, selecting the good ones for conservation and sowing, maintaining and cleaning the water ponds of invasive plants and manuring^32^. If these tasks are not properly completed, cross-breeding with wild species or self-pollination can occur as water chestnut seeds can lie dormant for years on the bottom of a body of water such as a lake or pond^30^. Thus, the finding that the majority of the Neolithic water chestnuts are attributed to Type III, and increase in size from layer 8 to 6, is strong evidence that they were already in the process of domestication and being actively selected and cultivated for desirable traits (size and shape of the fruit).

The Hemudu Culture, to which the Tianluoshan site is attributed, represents the transitional period from hunting-gathering to agriculture^10,33^. Like water chestnuts, vast quantities of rice were found at Tianluoshan and previous research determined that it was also under the process of domestication^34–36^. In particular, the discovery of rice fields around the dwellings indicates that the people had knowledge about the practices of rice agriculture such as: burning straw, weeding and watering the land^35^. The development and advances of agricultural practices during this period likely reflects an understanding of the conditions and activities that were beneficial to the cultivation of a wide variety of plants. Besides water chestnuts and rice, excavations at the Middle and Late Neolithic sites on the Lower Yangtze River Valley found the remains of many other edible plants (eg. peach, tea (*Camellia sinensis* L.) and melon (*Cucumis melo* L.)), and these are also believed to have been cultivated and possibly in the process of domestication^19,26,36–39^. Thus, being one of the essential food resources of the Hemudu Culture, it is not difficult to understand that water chestnuts were actively selected and cultivated by the Tianluoshan inhabitants, and constitute an important component to the early agricultural practices of the site.

Since the majority of the Tianluoshan water chestnuts unearthed from layer 8 already show a similar type with *Trapa bispinosa*, while only a minority are approximately close to *Trapa incisa*, this suggests that the domestication of water chestnuts in this region had commenced sometime earlier than approximately 7000 years ago. Thus, the Tianluoshan water chestnuts were already in the process of domestication but had not yet reached the modern version in terms of size and shape of the domesticated species. So far among the sites of the Shangshan Culture (11000–8600 cal BP), which is one of the oldest Neolithic cultures discovered in the Lower Yangtze River Valley, only starch grains of water chestnuts have been found^40^. However, the macro-remains of water chestnuts have been recovered at the Kuahuqiao site (8000–7000 cal BP)^41^ (Fig. 1b,c). Future work is planned to investigate the size and shape of the water chestnuts from Kuahuqiao, and these results will be compared to those of Tianluoshan to better understand the timing and the process of water chestnut cultivation and management in the Lower Yangtze River Valleys of China.

## Conclusions

In spite of being previously considered as a wild dietary resource that was gathered during the Neolithic^15,16^, the water chestnuts at Tianluoshan demonstrate that they were already under intensive domestication and cultivation by approximately 7000 BP. This finding supports the view that Neolithic humans had a good command and understanding of natural resources such as the growth cycles and requirements of various plant species. These findings at Tianluoshan support past archaeobotanical research^20–22,43,44^ from other parts of the world that agricultural intervention was applied to a series of plant resources, rather than to cereals only. Thus, humans had well-adapted and developed subsistence strategies based on local environmental and climate conditions. This viewpoint is now being recognized by more scholars in China^38,45–48^, and contributes to a deeper understanding of how non-cereal plants were influenced by human activity and selection in the Lower Yangtze River Valleys of China.

## Methods

Two types of methods were used to collect the water chestnuts at Tianluoshan. 1) In storage pits, the majority of water chestnuts were preserved intact and hand shovels were used to excavate and collect the remains. 2) In the cultural layers, the water chestnut remains were generally husk fragments. However, after manually washing the soil, some complete water chestnuts were selected from the other botanical remains with the use of a mesh screen (0.34 mm).

Complete or nearly complete water chestnuts (n = 481) from five different excavated grids (layers 6 to 8) of the Tianluoshan site were collected and measured with an electronic digital caliper (brand: Guanglu, range: 0–200 mm) to two significant figures (Fig. 2a,b). Since most of the Neolithic water chestnuts do not have obvious necks and humps, which are diagnostic characteristics of modern species, we decided to take measurements of length, height, thickness, top diameter and bottom diameter^49–51^ to quantify the shapes and sizes of the specimens (Fig. 2c).

The majority of the Neolithic samples (n = 384) were preserved in their original waterlogged condition. However, some of the water chestnuts (n = 97) were previously conserved in a solution of trehalose and water according to the protocol of Nagahama *et al*.^52^. In order to investigate possible differences related to these preservation conditions between waterlogged (n = 261) and trehalose preserved water chestnuts (n = 52), measurements from both types of samples from pit H69 (layer 6) are directly compared. In addition, eight different modern water chestnuts (n = 447) were also measured including three wild varieties (n = 268) and five domesticated types (n = 179) (Fig. 3). One of these wild populations grows freely inside of the Hemudu site park (~7 km from Tianluoshan), and the other two are from Hubei Province and Shandong Province. The domesticated species were purchased from markets in Yuyao, Jiaxing and Hangzhou, modern cities located in eastern and northern Zhejiang Province.

Water chestnuts were also sorted into three different shape classifications. These were characterized as: Type I (wild), Type II (intermediate) and Type III (domesticated) (Fig. 2a)^1,49–51^. The classification system developed here was based on past research which found that the size of the fruit is an extremely important criteria when classifying water chestnuts^49,50^. In addition, the shape and number of horns^2,49,50^, the existence and height of the mastoid^50^ and the shape of fruit^27,31^ can all function as features to separate water chestnuts into different groups. First, the water chestnuts at Tianluoshan were placed into two groups according to the size of the fruit. The first group (containing Type I and II specimens) is smaller in size, with an average length of less than 30 mm. In addition, the Type I and II water chestnuts display certain wild type characteristics, such as comparatively sharp horns (defense against predators and to help the water chestnuts insert into the bottom of lakes for reproduction), a short bottom diameter (allowing it to break from the stem easier), and a bigger crown (Fig. 2a–c). In contrast, the second group is composed of the Type III specimens, and these are bigger in size with the majority of samples longer than 30 mm. Further, the Type III water chestnuts display certain domesticated features such as relatively shorter, wider and rounded horns, a wide bottom diameter and smaller or not obvious crowns (Fig. 2a–c).

After this initial classification, some additional differences in the first group suggest that they could be further divided into two additional types: Type I and Type II specimens. This was based on the work of Kadono^27^ who pointed out that the shape of the fruit is a critical feature when classifying *Trapa* and Wan^51^ who argued that the mastoid plays an important role in the classification of water chestnuts. Visual observation of the water chestnuts in the first group found that some of the samples had horns that slant upward, making the whole water chestnut a narrow inverted triangle shape (Fig. 2a (wild)). In addition, these specimens have mastoids on their abdomen. As these characteristics are quite similar to the modern wild water chestnuts collected in this paper, these specimens were classified as Type I (wild). The rest of the samples were observed to have horizontal horns, a diamond shape and few mastoids on their abdomen. These specimens display differences compared to the modern wild varieties, but have not reached the shape and size of the domesticated samples. Therefore, these specimens were classified as Type II or and intermediate stage of development.

### Data availability

All data generated or analysed during this study are included in this published article (and its Supplementary Information files).

## Electronic supplementary material

Supplementary Information

## References

[1] Flora Commitment of Chinese Academy of Sciences. *Flora of China* 3–27 (China Science Publishing, 2000).

[2] Peng, J. *et al*. The review on research of water chestnuts in China. *Nat. Aquat. Veg. Res*. supplementary issue, 76–80 (2007).

[3] Xia RB (1996). Cultivation and utilization of the historic water chestnuts in the southern Yangtze River Valley. Agric. His. of Chin..

[4] Zhu, D. W., Wang, D. B. & Li, X. X. (ed.) *Chinese crops and their kindred plants: vegetables* 1107–1120 (China Agriculture Press, 2008).

[5] Tang XT, Zheng FS, Qin J, Lu MX, Du YZ (2016). Genetic Structure of Water Chestnut Beetle: Providing Evidence for Origin of Water Chestnut. Plos One.

[6] Karg S (2006). The water chestnut (Trapa natans L.) as a food resource during the 4^th^ to 1^st^ millennia BC at Lake Federsee, Bad Buchau (southern Germany). Environmental Archaeology.

[7] Borojevic, K. Water chestnuts (Trapa natans L.) as controversial plants: Botanical, ethno-historical and archaeological evidence In: Fairbairn, A. & Weiss, E. (eds.) From Foragers to Farmers. Papers in Honor of Gordon C. Hillman. Oxford, 86–97 (2009).

[8] Yu WJ (1992). Discussion on water chestnuts unearthed at the Hemudu site. Agri. Archaeol..

[9] You XL (1993). Stories on water chestnuts. Ancient and Modern Agric..

[10] Qin, L., Fuller, D. Q. & Harvey, E. Subsistence of Hemudu Site, and reconsideration of issues in the study of early rice from Lower Yangzte in *Dongfang Kaogu [Oriental Archaeology]* 3 (ed. Archaeology Research Center of Shandong University) 307–350 (Science Press, 2006).

[11] Yu, W. J. Eat rice, wear flax: dress and diet culture of the Liangzhu people 62–66 (Zhejiang Photography Press, 2007).

[12] Pan, Y. Resource production in the Yangtze River Delta Region and Qiantang River Region in 10000-6000 BP. PhD. thesis, Fudan University (2011).

[13] Fuller, D. Q. *et al*. Archaeobotanical analysis of the plants at the Tianluoshan site in *Integrated Studies on the Natural Remains at* Tianluoshan (ed. Zhejiang Provincial Institute of Cultural Relics and Archaeology & Chinese Archaeology Research Center of Peking University) 47–96 (Cultural Relics Press, 2011).

[14] Zhao ZJ (2014). The process of origin of agriculture in China: archaeological evidence from flotation results. Quaternary Sci..

[15] Zhao ZJ, Zhang JZ (2009). Analysis report of the flotation at the Jiahu site in 2001. Archaeology.

[16] Zhang C (2011). The cultural remains of the period one of the Jiahu site. Cultural Relics.

[17] Ningbo Municipal Cultural Relics and Archaeological Research Institute. *Fujiashan: excavation report of the Neolithic site* 144–160 (Science Press, 2013).

[18] Sun GP, Huang WJ (2007). Brief report of the excavation on a Neolithic site at Tianluoshan Hill in Yuyao City, Zhejiang. Cultural Relics.

[19] Sun, G. P. The brief introduction of the first period excavation at the Tianluoshan site (2004–2008) In *Integrated Studies on the Natural Remains at* Tianluoshan (ed. Zhejiang Provincial Institute of Cultural Relics and Archaeology & Peking University) 7–39 (Cultural Relics Press, 2011).

[20] Fairbairn A (2002). *Macro-botanical evidence for plant us*e at Neolithic Catalhöyük, southern-central Anatolia, Turkey. Vegetation History & Archaeobotany.

[21] Martinoli D, Jacomet S (2004). Identifying endocarp remains and exploring their use at Epipalaeolithic Öküzini in Southwest Anatolia, Turkey. Vegetation History & Archaeobotany.

[22] Weiss E, Kislev M, Hartmann A (2006). Autonomous cultivation before domestication. Science.

[23] Jacomet S (2006). Plant Economy of the Northern Alpine Lake Dwelling area − 3500-2400 BC cal. Environmental Archaeology.

[24] Antolín F (2016). Quantitative approximation to large-seeded wild fruit use in a late Neolithic lake dwelling. The case study of layer 13 of Parkhaus-Opéra in Zürich (Central Switzerland). Quaternary International.

[25] Jacomet S (2016). On-site data cast doubts on the hypothesis of shifting cultivation in the Late Neolithic (c. 4300-2400cal. BC): Landscape management as an alternative paradigm. The Holocene.

[26] Zheng, Y. F., Chen, X. G. & Sun, G. P. Plant seeds unearthed at Tianluoshan and the food production reflected in *Integrated Studies on the Natural Remains at* Tianluoshan (ed. Zhejiang Provincial Institute of Cultural Relics and Archaeology & Chinese Archaeology Research Center of Peking University) 97–107 (Cultural Relics Press, 2011).

[27] Guan SF, Lang Q (1987). New species of Trapa and Najas from Jiangxi, China. Bull. of Bot. Res..

[28] Song XZ, Hu JQ, Fang ZD (1981). Discussion on biological features and cultivation technology of Nanhu water chestnuts (in Chinese). *Journal of Zhejiang Agric*. Sci..

[29] Wang LJ, Ding BY (1997). Chromosome analysis on three kinds of water chestnuts in China (in Chinese). Ningbo Agric. Sci. and Tech..

[30] Ding BY, Hu RY, Shi MZ, Zheng CZ (1996). A preliminary study on the pollination biology of *Trapa* L. (in Chinese). J. of Hangzhou Univ. (Nat. Sci.).

[31] Kadono Y (1987). A preliminary study on the variation of *Trapa* in Japan (in Japanese). Acta Phyto. Geobot..

[32] Yu, Q. S., Dong, X. Y & Zhong, L. F. *The Methods of Growing Water Chestnuts* (Science and Technology of China Press, 1958).

[33] Zhao ZJ (2011). New Archaeobotanic Data for the Study of the Origins of Agriculture in China (in Chinese). Current Anthropol..

[34] Fuller DQ (2009). The domestication process and domestication rate in rice: spikelet bases from the Lower Yangtze. Science.

[35] Li CH, Zheng. YF, Yu SY, Li YX, Shen HD (2012). Understanding the ecological background of rice agriculture on the Ningshao Plain during the Neolithic Age: pollen evidence from a buried paddy field at the Tianluoshan cultural site. Quaternary Sci. Rev..

[36] Zheng YF (2009). Rice fields and modes of rice cultivation between 5000 and 2500 BC in east China. J. of Archaeol. Sci..

[37] Fuller DQ, Hosoya LA, Zheng YF, Qin L (2010). A Contribution to the Prehistory of Domesticated Bottle Gourds in Asia: Rind Measurements from Jomon Japan and Neolithic Zhejiang, China. Econ. Bot..

[38] Zheng YF, Crawford GW, Chen XG (2014). Archaeological Evidence for Peach *(Prunus persic*a) Cultivation and Domestication in China. Plos One.

[39] Li, R. R., Yang, M. & Zhu, Q. L. 6000 BP Tea tree remains cultivated by humans is Discovered at the Tianluoshan site, Yuyao, http://www.chinanews.com/df/2015/06-30/7375772.shtml (2015).

[40] Zhejiang Provincial Research Institute of Cultural Relics and Archaeology & Pujiang Museum. Pujiang Shangshan 274 (Cultural Relics Press, 2016).

[41] Zhejiang Provincial Research Institute of Cultural Relics and Archaeology. Kuahuqiao (Cultural Relics Press, 2004).

[42] Pei AP (2008). Broad-spectrum Economy and Rice Agriculture in Prehistory (in Chinese). Agric. His. of China.

[43] Martinoli D, Nesbitt M (2003). Plant stores at pottery Neolithic Höyücek, southwest Turkey. Anatolian Studies.

[44] Martinoli D (2004). Food plant use, temporal changes and site seasonality at Epipalaeolithic Öküzini and Karain B Caves, Southwest Anatolia, Turkey. Paléorient.

[45] Weiss E (2008). Plant-food preparation area on an Upper Palaeolithic brush hut floor at Ohalo II, Israel. Journal of Archaeological Science.

[46] Liu L (2010). The Exploitation of Acorn and Rice in Early Holocene Lower Yangzi River China. *Acta Anthropol*. Sinica.

[47] Crawford GW (2012). Early rice exploitation in the lower Yangzi valley: What are we missing?. The Holocene.

[48] Yang XY (2015). Barnyard grasses were processed with rice around 10000 years ago. Sci. Rep..

[49] Wang YF, Ding BY, Hu RY, Jin ML (2006). Analysis of morphological plasticity of Trapa from China and its taxonomic signification (in Chinese). J. of Zhejiang Univ. (Sci. Edition).

[50] Hu RY, Ding BY (2001). A numerical taxonomic study of Trapa from China (in Chinese). J. of Zhejiang Univ. (Agric. and Life Sci.).

[51] Wan WH (1984). The classification research on Trapaceae in China (in Chinese). J. of Nanchang Univ. (Nat. Sci.).

[52] Nagahama K, Uchiyama N, Nakamura K (2012). Report on the Trehalohse Method for the Conservation of Archaeological Waterlogged Wood (in Japanese). From Jomon No Mori.

[53] Liu, C. J., Jin, G. Y. & Kong, Z. C. *Archaeobotany: research on seeds and fruits* (Science Press, 2008).

[54] Pei AP (1998). A reconsideration on rice remains of Pengtoushan Culture and the rice agriculture in prehistoric China (in Chinese). Agric. Archaeol..

[55] Zhejiang Provincial Cultural Relics Management Committee & Zhejiang Provincial Museum (1978). Report on the first period excavation of the Hemudu site. Acta Archaeol. Sinica.

[56] Zhang MK (1999). The report of trial excavation of the Xinqiao site at Tongxiang (in Chinese). Agric. Archaeol..

[57] Jiaxing Municipal Bureau of Culture. The Caoxieshan site in Wuxian, Jiangsu Province. *The Majiabang Culture* 126-133 (Zhejiang Photography Press, 2004).

[58] Zhu C (2000). Environmental archaeology on the Longqiuzhuang Neolithic site, Gaoyou, Jiangsu Province (in Chinese). J. of Nanjing Univ. (Nat. Sci.).

[59] Longqiuzhuang Archaeological Excavation Team. *Longqiuzhuang: excavation report of the Neolithic site in Jianghuai Region* 440–463 (Science Press, 1999).

[60] Gao, M. H. An environmental study on the lower reaches of the Yangtze River based on archaeological sequence: the ecological system and the relationship between human and environment between the process of civilization (in Chinese). PhD. thesis, Fudan University (2003).

[61] Wen HF (1993). The preliminary research on diet culture in pre-Qin Dynasty, Taihu District (in Chinese). Southeast Culture.

[62] Zhejiang Provincial Research Institute of Cultural Relics and Archaeology. *Bianjiashan* 406–422 (Cultural Relics Press, 2014).

[63] Ji ZQ (1983). A Neolithic site at Qingdun in Haian County, Jiangsu Province (in Chinese). Acta Archaeol. Sinica.

[64] Zhejiang Provincial Cultural Relics Management Committee. The report of the first and second excavation at the Qianshanyang site, Wuxing (in Chinese). *Acta Archaeol. Sinica* **2**, 1960.

[65] Zhejiang Provincial Research Institute of Cultural Relics and Archaeology, Huzhou Museum. *Qianshanyang (reports of the 3*^*rd*^*, 4*^*th*^*excavations)* (Cultural Relics Press, 2014).

[66] Guo, X. R. Research of prehistoric plant remains from Guangfulin Site in Shanghai (in Chinese). Master thesis, Shandong University (2014).

[67] An, Y. X. The plant use and early wetland exploration in the pre-Qin period of Guangfulin site (in Chinese). Master thesis, Shandong University (2014).

[68] Wu, X. H., Qin L. & Sun, G.P. Radiocarbon age data of the Tianluoshan site in *Integrated Studies on the Natural Remains at**Tianluoshan* (ed. Zhejiang Provincial Institute of Cultural Relics and Archaeology & Chinese Archaeology Research Center of Peking University) 40–46 Cultural Relics Press, (2011).

[69] Jin (2014). A primary study on AMS 14C dating of phytolith at Tianluoshan site, Zhejiang Province (in Chinese). Quaternary Sci..

