# Supplementary material for: Neolithic cultivation of water chestnuts (Trapa L.) at Tianluoshan (7000-6300 cal BP), Zhejiang Province, China
Source: Sci Rep. 2017 Nov 24;7:16206. doi: 10.1038/s41598-017-15881-w (PMC5701232; doi:10.1038/s41598-017-15881-w)
Supplement: Supplementary file 1 — Supplementary Information [file 41598_2017_15881_MOESM1_ESM.doc]

# Supplementary Information for:

**Neolithic cultivation of water chestnuts (*Trapa* L.) at Tianluoshan (7000-6300 cal BP), Zhejiang Province, China**

Yi Guo^1†*^, Rubi Wu^1†*^, Guoping Sun^2^, Yunfei Zheng^2^, Benjamin T. Fuller^3^

*^1^Department of Cultural Heritage and Museology, School of Humanities, Zhejiang University, Hangzhou, 310028, China*

*^2^ Zhejiang Provincial Research Institute of Cultural Relics and Archaeology, Hangzhou, 310014, China*

*^3^Department of Archaeology and Anthropology, University of Chinese Academy of Sciences, Beijing 100049, China*

†=Co-first author

*=Address for correspondence:

e-mail: [guoyi10@zju.edu.cn](mailto:guoyi10@zju.edu.cn)

rubiwu@zju.edu.cn

Supplementary Table 1 Modern Samples

| **Sample** | **Length (mm)** | **Height (mm)** | **Thickness (mm)** | **Top Diameter (mm)** | **Bottom Diameter (mm)** | **Comments** | |
| --- | --- | --- | --- | --- | --- | --- | --- |
| Hemudu Modern Wild | 16.13 | 17.99 | 8.29 | 4.03 | 6.03 | *Trapa incisa*, collected from the Hemudu site park, Yuyao, Zhejiang Province, China | |
| Hemudu Modern Wild | 15.64 | 16.45 | 8.28 | 4.52 | 4.32 |
| Hemudu Modern Wild | 16.29 | 14.55 | 7.51 | 4.96 | 3.58 |
| Hemudu Modern Wild | 16.00 | 15.80 | 8.30 | 5.17 | 3.52 |
| Hemudu Modern Wild | 17.51 | 17.29 | 8.44 | 5.43 | 4.53 |
| Hemudu Modern Wild | 18.38 | 14.29 | 8.20 | 4.83 | 3.85 |
| Hemudu Modern Wild | 13.15 | 15.71 | 7.61 | 3.36 | 4.37 |
| Hemudu Modern Wild | 15.60 | 15.23 | 8.44 | 5.08 | 3.89 |
| Hemudu Modern Wild | 17.15 | 13.28 | 8.33 | 2.71 | 3.66 |
| Hemudu Modern Wild | 15.53 | 16.02 | 8.20 | 3.32 | 3.81 |
| Hemudu Modern Wild | 16.43 | 16.50 | 8.85 | 4.86 | 4.44 |
| Hemudu Modern Wild | 16.87 | 15.45 | 8.64 | 3.38 | 3.84 |
| Hemudu Modern Wild | 16.15 | 15.45 | 8.20 | 3.88 | 3.87 |
| Hemudu Modern Wild | 15.39 | 13.18 | 8.17 | 4.41 | 2.85 |
| Hemudu Modern Wild | 16.92 | 14.17 | 8.55 | 4.72 | 3.22 |
| Hemudu Modern Wild | 14.38 | 15.37 | 7.67 | 3.92 | 3.94 |
| Hemudu Modern Wild | 17.01 | 16.96 | 8.79 | 4.81 | 4.62 |
| Hemudu Modern Wild | 17.80 | 14.97 | 8.28 | 4.62 | 3.53 |
| Hemudu Modern Wild | 17.95 | 16.66 | 9.11 | 5.43 | 3.74 |
| Hemudu Modern Wild | 15.59 | 14.29 | 8.21 | 4.40 | 3.79 |
| Hemudu Modern Wild | 14.05 | 17.21 | 8.51 | 3.61 | 3.74 |
| Hemudu Modern Wild | 18.47 | 17.36 | 9.06 | 4.20 | 5.62 |
| Hemudu Modern Wild | 17.03 | 16.22 | 8.59 | 3.99 | 4.58 |
| Hemudu Modern Wild | 17.48 | 16.70 | 9.81 | 4.76 | 4.54 |
| Hemudu Modern Wild | 14.95 | 16.20 | 8.79 | 4.07 | 4.44 |
| Hemudu Modern Wild | 18.29 | 17.76 | 10.09 | 5.78 | 4.96 |
| Hemudu Modern Wild | 18.28 | 16.71 | 9.19 | 5.32 | 4.58 |
| Hemudu Modern Wild | 14.08 | 18.53 | 8.71 | 3.78 | 4.53 |
| Hemudu Modern Wild | 16.84 | 15.38 | 8.44 | 4.40 | 3.84 |
| Hemudu Modern Wild | 14.47 | 15.80 | 9.00 | 4.72 | 4.47 |
| Hemudu Modern Wild | 14.11 | 14.28 | 7.59 | 2.91 | 4.15 |
| Hemudu Modern Wild | 15.52 | 17.57 | 9.15 | 4.12 | 5.64 |
| Hemudu Modern Wild | 14.46 | 14.17 | 8.09 | 3.70 | 3.83 |
| Hemudu Modern Wild | 17.32 | 17.79 | 9.53 | 5.74 | 4.52 |
| Hemudu Modern Wild | 15.25 | 15.73 | 8.22 | 4.72 | 4.52 |
| Hemudu Modern Wild | 13.89 | 12.23 | 6.68 | 3.53 | 2.92 |
| Hemudu Modern Wild | 17.62 | 16.16 | 8.92 | 4.33 | 4.05 |
| **Mean** | 16.16 | 15.82 | 8.50 | 4.37 | 4.17 |  | |
| **±SD** | 1.46 | 1.46 | 0.65 | 0.76 | 0.68 |  | |
| **Number** | 37 | 37 | 37 | 37 | 37 |  | |
| **Sample** | **Length (mm)** | **Height (mm)** | **Thickness (mm)** | **Top Diameter (mm)** | **Bottom Diameter (mm)** | **Comments** |  |
| Hubei Wild | 16.13 | 17.20 | 13.85 | 4.32 | 8.55 | *Trapa incisa*(?), purchased online, grows freely in Zhangjia Lake, Tianmen, Hubei Province, China |  |
| Hubei Wild | 18.94 | 17.99 | 14.58 | 4.83 | 10.68 |  |
| Hubei Wild | 18.90 | 16.29 | 15.48 | 4.95 | 9.50 |  |
| Hubei Wild | 17.92 | 18.52 | 13.21 | 5.24 | 10.8 |  |
| Hubei Wild | 19.84 | 17.15 | 14.60 | 5.36 | 8.68 |  |
| Hubei Wild | 20.39 | 18.61 | 14.85 | 4.07 | 11.45 |  |
| Hubei Wild | 18.47 | 16.08 | 13.73 | 5.00 | 8.05 |  |
| Hubei Wild | 19.20 | 18.94 | 16.48 | 4.91 | 11.15 |  |
| Hubei Wild | 20.38 | 16.98 | 14.09 | 4.68 | 8.67 |  |
| Hubei Wild | 19.54 | 19.02 | 15.62 | 5.49 | 11.34 |  |
| Hubei Wild | 17.37 | 16.06 | 13.66 | 5.62 | 10.11 |  |
| Hubei Wild | 17.10 | 15.48 | 14.48 | 5.47 | 9.24 |  |
| Hubei Wild | 19.76 | 15.75 | 14.33 | 5.20 | 9.03 |  |
| Hubei Wild | 17.14 | 15.89 | 13.94 | 5.52 | 9.63 |  |
| Hubei Wild | 20.70 | 16.21 | 13.32 | 5.41 | 10.47 |  |
| Hubei Wild | 18.73 | 18.16 | 14.46 | 4.88 | 10.56 |  |
| Hubei Wild | 20.58 | 19.20 | 15.81 | 5.21 | 10.26 |  |
| Hubei Wild | 20.08 | 17.44 | 13.40 | 5.61 | 10.65 |  |
| Hubei Wild | 21.78 | 14.20 | 13.54 | 4.82 | 7.54 |  |
| Hubei Wild | 18.24 | 13.38 | 11.68 | 4.65 | 7.06 |  |
| Hubei Wild | 20.56 | 16.54 | 14.58 | 4.93 | 9.64 |  |
| Hubei Wild | 19.54 | 17.09 | 13.42 | 4.56 | 10.2 |  |
| Hubei Wild | 18.03 | 17.99 | 15.17 | 4.86 | 10.08 |  |
| Hubei Wild | 19.36 | 14.59 | 12.44 | 5.06 | 8.40 |  |
| Hubei Wild | 23.93 | 18.09 | 14.96 | 5.81 | 10.32 |  |
| Hubei Wild | 22.36 | 17.08 | 14.62 | 4.88 | 10.16 |  |
| Hubei Wild | 19.67 | 19.25 | 15.27 | 5.32 | 10.94 |  |
| Hubei Wild | 18.50 | 18.24 | 12.80 | 4.96 | 10.01 |  |
| Hubei Wild | 20.04 | 17.01 | 14.65 | 4.80 | 9.80 |  |
| Hubei Wild | 18.26 | 16.52 | 13.35 | 6.33 | 10.61 |  |
| Hubei Wild | 22.37 | 16.29 | 13.66 | 4.63 | 10.19 |  |
| Hubei Wild | 13.95 | 14.16 | 13.04 | 4.99 | 8.49 |  |
| Hubei Wild | 19.01 | 18.38 | 14.50 | 4.23 | 12.07 |  |
| Hubei Wild | 20.10 | 14.80 | 13.74 | 5.56 | 8.73 |  |
| Hubei Wild | 24.54 | 18.69 | 15.89 | 4.87 | 11.83 |  |
| Hubei Wild | 22.21 | 18.86 | 15.46 | 5.75 | 10.37 |  |
| Hubei Wild | 26.61 | 19.14 | 15.15 | 6.76 | 11.59 |  |
| Hubei Wild | 20.16 | 15.44 | 16.29 | 5.34 | 9.99 |  |
| Hubei Wild | 18.68 | 16.42 | 13.05 | 4.55 | 9.14 |  |
| Hubei Wild | 22.45 | 16.24 | 16.51 | 5.02 | 9.73 |  |
| Hubei Wild | 17.60 | 16.06 | 14.67 | 5.07 | 7.85 |  |
| Hubei Wild | 21.87 | 15.90 | 14.76 | 5.03 | 9.59 |  |
| Hubei Wild | 21.50 | 15.08 | 14.74 | 5.48 | 8.48 |  |
| Hubei Wild | 22.59 | 16.30 | 15.83 | 5.34 | 10.43 |  |
| Hubei Wild | 23.76 | 19.02 | 18.45 | 5.22 | 13.61 |  |
| Hubei Wild | 38.15 | 16.72 | 14.49 | 6.38 | 11.42 |  |
| Hubei Wild | 23.63 | 15.41 | 15.51 | 5.38 | 7.34 |  |
| Hubei Wild | 25.06 | 16.87 | 13.38 | 5.01 | 10.43 |  |
| Hubei Wild | 24.00 | 16.35 | 15.53 | 5.31 | 9.60 |  |
| Hubei Wild | 23.52 | 15.24 | 13.57 | 5.29 | 9.42 |  |
| Hubei Wild | 22.76 | 15.8 | 15.18 | 5.38 | 7.80 |  |
| Hubei Wild | 21.22 | 16.18 | 13.69 | 5.57 | 9.38 |  |
| Hubei Wild | 22.97 | 16.76 | 16.00 | 5.66 | 11.03 |  |
| Hubei Wild | 20.76 | 17.67 | 14.34 | 5.12 | 10.79 |  |
| Hubei Wild | 22.63 | 18.98 | 15.47 | 5.44 | 12.13 |  |
| Hubei Wild | 23.53 | 18.40 | 16.76 | 4.18 | 12.42 |  |
| Hubei Wild | 19.97 | 16.50 | 14.52 | 4.31 | 10.01 |  |
| Hubei Wild | 24.09 | 17.52 | 15.76 | 4.71 | 12.02 |  |
| Hubei Wild | 20.84 | 16.60 | 13.64 | 4.51 | 8.97 |  |
| Hubei Wild | 19.45 | 16.30 | 12.57 | 5.10 | 9.33 |  |
| Hubei Wild | 19.79 | 18.53 | 13.88 | 3.77 | 11.16 |  |
| Hubei Wild | 21.01 | 16.80 | 13.16 | 4.94 | 9.88 |  |
| Hubei Wild | 19.43 | 16.80 | 12.56 | 4.85 | 8.56 |  |
| Hubei Wild | 23.71 | 15.73 | 13.26 | 5.36 | 9.50 |  |
| Hubei Wild | 19.74 | 14.79 | 12.47 | 4.91 | 7.48 |  |
| Hubei Wild | 19.43 | 17.92 | 12.51 | 4.19 | 9.72 |  |
| Hubei Wild | 19.95 | 15.91 | 12.52 | 4.71 | 10.16 |  |
| Hubei Wild | 23.74 | 21.72 | 15.69 | 6.53 | 12.73 |  |
| Hubei Wild | 20.38 | 18.15 | 13.19 | 4.22 | 8.57 |  |
| Hubei Wild | 20.19 | 17.67 | 13.33 | 4.28 | 9.83 |  |
| Hubei Wild | 20.11 | 15.59 | 12.61 | 5.04 | 7.66 |  |
| Hubei Wild | 20.57 | 14.58 | 12.75 | 5.33 | 9.09 |  |
| Hubei Wild | 19.97 | 15.87 | 13.27 | 4.93 | 7.45 |  |
| Hubei Wild | 22.14 | 16.77 | 13.43 | 4.47 | 7.28 |  |
| Hubei Wild | 22.22 | 18.01 | 14.22 | 4.78 | 10.91 |  |
| Hubei Wild | 27.62 | 20.24 | 13.88 | 4.49 | 12.94 |  |
| Hubei Wild | 22.14 | 17.88 | 13.31 | 4.40 | 11.51 |  |
| Hubei Wild | 21.02 | 16.91 | 13.76 | 4.84 | 9.26 |  |
| Hubei Wild | 21.28 | 18.64 | 14.38 | 4.96 | 12.57 |  |
| Hubei Wild | 20.99 | 15.28 | 12.32 | 5.01 | 9.24 |  |
| Hubei Wild | 22.58 | 17.81 | 14.60 | 5.32 | 10.43 |  |
| Hubei Wild | 22.10 | 16.80 | 13.74 | 4.70 | 9.94 |  |
| Hubei Wild | 22.56 | 18.45 | 14.13 | 4.83 | 9.61 |  |
| Hubei Wild | 21.91 | 17.67 | 13.17 | 4.13 | 10.09 |  |
| Hubei Wild | 20.67 | 18.20 | 13.07 | 4.72 | 8.32 |  |
| Hubei Wild | 20.01 | 17.75 | 13.38 | 4.46 | 11.35 |  |
| Hubei Wild | 23.84 | 15.55 | 13.04 | 5.45 | 8.62 |  |
| Hubei Wild | 22.00 | 19.33 | 14.18 | 4.23 | 12.44 |  |
| Hubei Wild | 22.71 | 17.31 | 12.90 | 4.60 | 9.20 |  |
| Hubei Wild | 19.99 | 17.40 | 13.12 | 4.27 | 10.70 |  |
| Hubei Wild | 21.73 | 16.29 | 13.85 | 4.79 | 10.89 |  |
| Hubei Wild | 20.67 | 14.36 | 12.24 | 4.79 | 8.05 |  |
| Hubei Wild | 22.17 | 15.56 | 12.60 | 5.50 | 7.61 |  |
| Hubei Wild | 18.90 | 15.54 | 11.65 | 4.81 | 10.31 |  |
| Hubei Wild | 19.67 | 15.69 | 12.05 | 5.16 | 7.62 |  |
| Hubei Wild | 21.55 | 15.80 | 12.28 | 4.87 | 9.88 |  |
| Hubei Wild | 21.41 | 15.91 | 12.82 | 4.98 | 9.43 |  |
| Hubei Wild | 23.46 | 14.84 | 12.02 | 4.99 | 7.82 |  |
| Hubei Wild | 22.38 | 15.65 | 12.93 | 5.27 | 9.51 |  |
| Hubei Wild | 20.81 | 18.04 | 13.43 | 4.36 | 11.16 |  |
| Hubei Wild | 20.77 | 13.84 | 11.87 | 5.05 | 7.45 |  |
| Hubei Wild | 22.75 | 17.23 | 13.40 | 4.64 | 9.95 |  |
| Hubei Wild | 24.26 | 16.61 | 13.13 | 4.50 | 9.65 |  |
| Hubei Wild | 22.05 | 16.16 | 12.29 | 4.93 | 8.92 |  |
| Hubei Wild | 22.56 | 17.50 | 14.09 | 5.02 | 12.09 |  |
| Hubei Wild | 23.98 | 17.54 | 13.55 | 5.03 | 11.08 |  |
| Hubei Wild | 22.77 | 15.99 | 14.51 | 4.69 | 9.07 |  |
| Hubei Wild | 22.40 | 18.80 | 13.68 | 5.43 | 11.65 |  |
| Hubei Wild | 20.87 | 16.21 | 10.77 | 4.82 | 7.53 |  |
| Hubei Wild | 20.96 | 17.89 | 14.27 | 3.79 | 10.94 |  |
| Hubei Wild | 23.70 | 17.21 | 13.37 | 5.63 | 10.45 |  |
| Hubei Wild | 23.24 | 17.81 | 13.56 | 4.86 | 10.28 |  |
| **Mean** | 21.23 | 16.91 | 13.92 | 4.99 | 9.87 |  |  |
| **±SD** | 2.69 | 1.46 | 1.26 | 0.52 | 1.42 |  |  |
| **Number** | 112 | 112 | 112 | 112 | 112 |  |  |
| **Sample** | **Length (mm)** | **Height (mm)** | **Thickness (mm)** | **Top Diameter (mm)** | **Bottom Diameter (mm)** | **Comments** | |
| Shandong Wild | 20.88 | 18.15 | 12.79 | 4.18 | 5.43 | *Trapa incisa*, purchased online, grows freely in Weishan Lake, Zaozhuang, Shandong Province, China | |
| Shandong Wild | 23.38 | 15.35 | 13.16 | 5.14 | 8.46 |
| Shandong Wild | 23.14 | 16.89 | 11.79 | 5.65 | 7.36 |
| Shandong Wild | 22.77 | 15.77 | 13.02 | 5.55 | 6.02 |
| Shandong Wild | 22.61 | 20.61 | 13.65 | 4.97 | 8.19 |
| Shandong Wild | 21.31 | 15.22 | 11.25 | 4.96 | 7.79 |
| Shandong Wild | 24.26 | 15.99 | 12.34 | 5.26 | 9.42 |
| Shandong Wild | 20.27 | 16.23 | 11.73 | 5.43 | 6.81 |
| Shandong Wild | 20.36 | 16.38 | 11.51 | 4.45 | 6.48 |
| Shandong Wild | 19.29 | 13.28 | 9.75 | 4.33 | 6.04 |
| Shandong Wild | 22.52 | 17.82 | 13.45 | 4.91 | 8.73 |
| Shandong Wild | 20.85 | 16.00 | 13.06 | 4.89 | 7.15 |
| Shandong Wild | 21.51 | 19.22 | 12.15 | 4.94 | 9.83 |
| Shandong Wild | 21.66 | 16.15 | 11.24 | 3.74 | 8.03 |
| Shandong Wild | 20.78 | 15.45 | 11.2 | 4.87 | 4.70 |
| Shandong Wild | 18.79 | 16.18 | 12.05 | 4.47 | 5.38 |
| Shandong Wild | 13.49 | 10.47 | 7.82 | 4.72 | 4.96 |
| Shandong Wild | 24.06 | 15.69 | 11.76 | 4.99 | 7.95 |
| Shandong Wild | 17.01 | 14.45 | 11.5 | 4.33 | 4.69 |
| Shandong Wild | 23.29 | 16.26 | 12.76 | 5.20 | 8.66 |
| Shandong Wild | 20.49 | 14.30 | 10.2 | 5.32 | 6.25 |
| Shandong Wild | 21.91 | 18.77 | 12.77 | 4.49 | 10.06 |
| Shandong Wild | 23.22 | 14.87 | 11.55 | 6.39 | 6.84 |
| Shandong Wild | 21.44 | 15.99 | 11.51 | 5.51 | 6.65 |
| Shandong Wild | 25.17 | 14.65 | 10.03 | 4.64 | 6.85 |
| Shandong Wild | 20.73 | 15.09 | 12.61 | 5.65 | 6.75 |
| Shandong Wild | 20.47 | 15.11 | 12.56 | 5.03 | 8.24 |
| Shandong Wild | 18.64 | 12.47 | 10.95 | 5.34 | 6.22 |
| Shandong Wild | 22.27 | 14.38 | 10.89 | 5.47 | 7.39 |
| Shandong Wild | 18.04 | 18.26 | 12.54 | 6.19 | 8.40 |
| Shandong Wild | 18.45 | 13.84 | 11.20 | 5.59 | 6.71 |
| Shandong Wild | 16.03 | 17.03 | 12.63 | 5.10 | 7.71 |
| Shandong Wild | 19.58 | 16.41 | 12.67 | 6.18 | 7.18 |  | |
| Shandong Wild | 15.82 | 15.69 | 11.55 | 5.75 | 7.32 |
| Shandong Wild | 17.80 | 15.69 | 11.44 | 4.63 | 8.25 |
| Shandong Wild | 18.59 | 16.63 | 11.86 | 4.20 | 8.32 |
| Shandong Wild | 18.01 | 16.93 | 11.56 | 4.84 | 7.70 |
| Shandong Wild | 14.14 | 17.94 | 11.10 | 4.77 | 9.03 |
| Shandong Wild | 17.26 | 17.71 | 11.81 | 4.89 | 6.47 |
| Shandong Wild | 18.05 | 14.97 | 11.44 | 5.32 | 6.30 |
| Shandong Wild | 23.98 | 16.19 | 14.50 | 5.18 | 10.57 |
| Shandong Wild | 17.98 | 17.73 | 12.26 | 4.21 | 8.06 |
| Shandong Wild | 17.32 | 17.08 | 11.46 | 4.65 | 7.65 |
| Shandong Wild | 17.92 | 16.71 | 11.18 | 4.79 | 7.00 |
| Shandong Wild | 19.12 | 17.09 | 12.36 | 4.49 | 9.11 |
| Shandong Wild | 18.67 | 17.67 | 11.37 | 4.46 | 10.83 |
| Shandong Wild | 19.30 | 17.53 | 13.05 | 5.49 | 7.79 |
| Shandong Wild | 20.36 | 14.62 | 10.49 | 5.19 | 7.13 |
| Shandong Wild | 22.80 | 19.64 | 11.73 | 4.71 | 9.01 |
| Shandong Wild | 20.67 | 16.84 | 12.74 | 4.27 | 10.45 |
| Shandong Wild | 16.56 | 14.21 | 10.11 | 5.32 | 6.30 |
| Shandong Wild | 17.29 | 16.26 | 11.13 | 4.67 | 7.48 |
| Shandong Wild | 19.51 | 13.01 | 10.62 | 4.55 | 6.26 |
| Shandong Wild | 15.11 | 16.67 | 11.70 | 5.01 | 6.69 |
| Shandong Wild | 15.31 | 15.23 | 11.11 | 4.81 | 6.67 |
| Shandong Wild | 17.24 | 18.97 | 11.26 | 4.16 | 7.24 |
| Shandong Wild | 15.88 | 15.54 | 10.57 | 4.64 | 7.87 |
| Shandong Wild | 20.45 | 19.33 | 12.65 | 5.40 | 8.59 |
| Shandong Wild | 19.67 | 16.94 | 10.91 | 5.23 | 7.51 |
| Shandong Wild | 17.15 | 14.63 | 10.85 | 5.31 | 6.76 |
| Shandong Wild | 21.83 | 19.47 | 14.52 | 4.86 | 10.43 |
| Shandong Wild | 19.93 | 14.95 | 11.52 | 5.51 | 6.71 |
| Shandong Wild | 21.28 | 18.7 | 13.89 | 4.49 | 6.63 |
| Shandong Wild | 21.53 | 14.7 | 10.79 | 5.71 | 6.66 |
| Shandong Wild | 20.54 | 15.94 | 11.66 | 5.42 | 8.18 |
| Shandong Wild | 25.64 | 20.75 | 14.88 | 5.55 | 10.01 |
| Shandong Wild | 15.99 | 17.94 | 13.59 | 4.15 | 6.03 |
| Shandong Wild | 21.41 | 18.76 | 12.94 | 4.98 | 10.64 |
| Shandong Wild | 18.84 | 20.41 | 14.82 | 4.73 | 6.79 |
| Shandong Wild | 21.58 | 17.15 | 11.44 | 5.52 | 9.11 |
| Shandong Wild | 25.87 | 17.18 | 11.01 | 3.97 | 9.41 |
| Shandong Wild | 23.12 | 18.48 | 13.68 | 5.23 | 8.14 |
| Shandong Wild | 19.72 | 16.07 | 12.48 | 5.44 | 9.02 |
| Shandong Wild | 18.2 | 17.45 | 12.45 | 5.56 | 8.21 |
| Shandong Wild | 22.59 | 16.79 | 12.63 | 4.59 | 9.93 |
| Shandong Wild | 21.21 | 17.31 | 11.60 | 5.82 | 8.76 |
| Shandong Wild | 16.91 | 14.91 | 9.62 | 4.38 | 7.17 |
| Shandong Wild | 19.28 | 16.98 | 11.00 | 4.66 | 7.34 |
| Shandong Wild | 16.86 | 16.89 | 11.73 | 4.45 | 4.42 |
| Shandong Wild | 18.11 | 16.91 | 10.62 | 4.76 | 7.99 |
| Shandong Wild | 21.91 | 16.54 | 11.45 | 4.65 | 8.81 |
| Shandong Wild | 18.01 | 16.65 | 11.72 | 5.16 | 8.22 |
| Shandong Wild | 16.97 | 15.53 | 11.8 | 4.27 | 4.25 |
| Shandong Wild | 16.94 | 10.80 | 9.18 | 4.71 | 5.53 |
| Shandong Wild | 17.91 | 17.20 | 12.18 | 5.61 | 7.57 |
| Shandong Wild | 18.82 | 17.67 | 13.05 | 5.39 | 7.99 |
| Shandong Wild | 21.19 | 18.3 | 12.43 | 8.00 | 10.77 |
| Shandong Wild | 16.96 | 19.21 | 12.65 | 5.04 | 8.18 |
| Shandong Wild | 17.99 | 17.6 | 14.06 | 4.29 | 4.41 |
| Shandong Wild | 24.10 | 17.19 | 11.74 | 4.27 | 8.96 |
| Shandong Wild | 20.82 | 17.46 | 10.9 | 5.45 | 8.21 |
| Shandong Wild | 21.43 | 16.49 | 11.67 | 3.69 | 6.30 |
| Shandong Wild | 18.43 | 17.19 | 12.07 | 5.45 | 8.66 |
| Shandong Wild | 22.69 | 15.38 | 12.43 | 5.94 | 7.30 |
| Shandong Wild | 19.68 | 17.35 | 11.5 | 4.32 | 7.43 |
| Shandong Wild | 18.78 | 16.89 | 12.24 | 4.6 | 7.06 |
| Shandong Wild | 22.34 | 17.84 | 11.89 | 4.39 | 10.13 |
| Shandong Wild | 17.95 | 15.15 | 10.87 | 6.27 | 8.34 |
| Shandong Wild | 20.89 | 14.35 | 11.17 | 5.17 | 6.19 |
| Shandong Wild | 15.71 | 15.69 | 10.91 | 4.40 | 7.19 |
| Shandong Wild | 21.45 | 16.95 | 11.42 | 4.35 | 9.63 |
| Shandong Wild | 20.85 | 17.19 | 12.50 | 4.67 | 6.55 |
| Shandong Wild | 26.13 | 19.79 | 13.47 | 4.85 | 9.82 |
| Shandong Wild | 23.29 | 18.53 | 12.54 | 4.47 | 11.57 |
| Shandong Wild | 25.77 | 17.78 | 12.38 | 4.75 | 10.87 |
| Shandong Wild | 19.32 | 15.61 | 11.88 | 4.98 | 8.02 |
| Shandong Wild | 21.94 | 17.58 | 12.75 | 5.28 | 6.89 |
| Shandong Wild | 22.08 | 17.43 | 11.94 | 6.12 | 8.81 |
| Shandong Wild | 25.65 | 18.86 | 12.62 | 3.99 | 9.03 |
| Shandong Wild | 17.34 | 14.36 | 10.94 | 4.90 | 7.65 |
| Shandong Wild | 30.68 | 17.38 | 12.57 | 5.05 | 9.35 |
| Shandong Wild | 19.46 | 16.84 | 10.41 | 5.10 | 6.78 |
| Shandong Wild | 19.4 | 17.11 | 10.95 | 4.24 | 8.67 |
| Shandong Wild | 18.94 | 16.21 | 13.27 | 5.55 | 8.24 |
| Shandong Wild | 24.09 | 16.51 | 11.96 | 5.05 | 8.61 |
| Shandong Wild | 20.12 | 17.86 | 12.11 | 4.44 | 8.45 |
| Shandong Wild | 19.35 | 15.94 | 11.3 | 4.58 | 5.64 |
| Shandong Wild | 21.10 | 16.46 | 9.57 | 6.23 | 7.26 |
| Shandong Wild | 21.35 | 18.08 | 13.82 | 4.37 | 5.07 |
| **Mean** | 20.16 | 16.60 | 11.88 | 4.98 | 7.73 |
| **±SD** | 2.84 | 1.76 | 1.16 | 0.63 | 1.53 |
| **Number** | 119 | 119 | 119 | 119 | 119 |
| **Sample** | **Length (mm)** | **Height (mm)** | **Thickness (mm)** | **Top Diameter (mm)** | **Bottom Diameter (mm)** | **Comments** | |
| Yuyao Domestic | 48.12 | 33.85 | 30.62 | 13.10 | 13.09 | *Trapa bispinosa* Roxb., purchased at the market in Yuyao, Zhejiang Province, China | |
| Yuyao Domestic | 38.54 | 22.55 | 23.30 | 9.17 | 12.53 |
| Yuyao Domestic | 36.47 | 24.08 | 24.49 | 9.32 | 11.17 |
| Yuyao Domestic | 38.69 | 26.50 | 26.70 | 9.64 | 14.69 |
| Yuyao Domestic | 37.22 | 25.41 | 21.06 | 9.06 | 11.07 |
| Yuyao Domestic | 37.04 | 25.90 | 19.65 | 10.01 | 10.99 |
| Yuyao Domestic | 32.60 | 23.84 | 20.48 | 9.08 | 11.74 |
| Yuyao Domestic | 31.84 | 24.19 | 19.97 | 8.58 | 11.24 |
| Yuyao Domestic | 39.25 | 27.46 | 19.77 | 8.52 | 11.53 |
| Yuyao Domestic | 33.97 | 23.18 | 24.54 | 11.13 | 11.37 |
| Yuyao Domestic | 43.90 | 30.10 | 28.19 | 9.59 | 12.56 |
| Yuyao Domestic | 44.91 | 31.89 | 24.74 | 9.79 | 12.08 |
| Yuyao Domestic | 37.28 | 27.06 | 20.22 | 10.81 | 11.63 |
| Yuyao Domestic | 38.59 | 24.82 | 25.30 | 8.51 | 12.15 |
| Yuyao Domestic | 35.33 | 23.57 | 20.27 | 8.93 | 9.45 |
| Yuyao Domestic | 45.71 | 32.37 | 28.49 | 12.02 | 15.29 |
| Yuyao Domestic | 41.19 | 25.58 | 22.48 | 8.78 | 13.15 |
| Yuyao Domestic | 38.82 | 23.46 | 27.09 | 8.81 | 11.94 |
| Yuyao Domestic | 42.36 | 30.08 | 27.23 | 9.09 | 11.72 |
| Yuyao Domestic | 39.69 | 24.85 | 24.21 | 9.03 | 12.98 |
| Yuyao Domestic | 49.37 | 31.20 | 28.86 | 10.63 | 14.68 |
| Yuyao Domestic | 39.55 | 26.49 | 26.62 | 9.98 | 11.97 |
| Yuyao Domestic | 43.25 | 29.10 | 30.20 | 10.25 | 10.90 |
| Yuyao Domestic | 36.69 | 25.68 | 22.42 | 8.54 | 8.55 |
| Yuyao Domestic | 43.59 | 25.85 | 27.27 | 9.40 | 10.58 |
| Yuyao Domestic | 40.47 | 20.79 | 25.78 | 8.19 | 10.86 |
| Yuyao Domestic | 32.05 | 22.85 | 18.83 | 7.07 | 9.93 |
| Yuyao Domestic | 43.03 | 26.71 | 26.10 | 10.40 | 11.66 |
| Yuyao Domestic | 39.99 | 28.53 | 26.63 | 8.87 | 12.07 |
| Yuyao Domestic | 45.61 | 29.60 | 23.78 | 11.66 | 10.69 |
| Yuyao Domestic | 41.70 | 26.78 | 25.43 | 8.81 | 11.18 |
| Yuyao Domestic | 38.86 | 25.47 | 19.97 | 10.22 | 9.33 |
| Yuyao Domestic | 45.10 | 28.15 | 27.09 | 10.71 | 11.52 |
| Yuyao Domestic | 40.47 | 24.70 | 23.32 | 8.93 | 10.23 |
| Yuyao Domestic | 41.30 | 24.39 | 24.00 | 9.58 | 10.12 |
| Yuyao Domestic | 38.73 | 25.63 | 24.04 | 10.53 | 9.13 |
| Yuyao Domestic | 38.60 | 23.58 | 16.73 | 8.89 | 8.91 |
| Yuyao Domestic | 33.28 | 23.07 | 21.78 | 8.41 | 10.79 |
| Yuyao Domestic | 37.61 | 24.44 | 22.13 | 8.92 | 10.62 |
| Yuyao Domestic | 40.72 | 27.78 | 22.95 | 10.35 | 10.38 |
| Yuyao Domestic | 40.24 | 26.68 | 27.91 | 9.59 | 11.76 |
| Yuyao Domestic | 40.34 | 22.68 | 23.43 | 10.56 | 10.43 |
| Yuyao Domestic | 38.32 | 27.13 | 25.28 | 9.65 | 9.63 |
| Yuyao Domestic | 41.87 | 28.51 | 23.60 | 11.31 | 10.22 |
| Yuyao Domestic | 34.79 | 23.71 | 23.71 | 8.59 | 10.10 |
| Yuyao Domestic | 44.26 | 28.59 | 24.23 | 10.45 | 12.80 |
| Yuyao Domestic | 49.94 | 34.33 | 30.11 | 12.48 | 11.52 |
| Yuyao Domestic | 35.05 | 21.93 | 19.00 | 9.45 | 9.54 |
| Yuyao Domestic | 34.15 | 22.48 | 19.80 | 8.14 | 8.30 |
| Yuyao Domestic | 41.47 | 27.41 | 29.13 | 9.44 | 11.21 |
| Yuyao Domestic | 39.74 | 25.66 | 24.56 | 9.83 | 10.28 |
| Yuyao Domestic | 41.15 | 27.27 | 21.35 | 10.00 | 10.57 |
| Yuyao Domestic | 34.78 | 22.83 | 10.25 | 10.01 | 8.20 |
| Yuyao Domestic | 40.96 | 25.70 | 12.14 | 9.27 | 10.08 |
| Yuyao Domestic | 38.53 | 26.78 | 27.19 | 8.53 | 11.15 |
| Yuyao Domestic | 43.70 | 29.89 | 26.34 | 9.92 | 11.85 |
| Yuyao Domestic | 39.10 | 26.51 | 25.94 | 9.29 | 11.75 |
| Yuyao Domestic | 38.65 | 26.15 | 21.54 | 8.66 | 11.08 |
| Yuyao Domestic | 40.15 | 28.08 | 26.18 | 9.95 | 10.37 |
| Yuyao Domestic | 44.40 | 28.21 | 27.76 | 10.32 | 10.89 |
| Yuyao Domestic | 41.30 | 24.40 | 22.96 | 9.10 | 9.73 |
| Yuyao Domestic | 37.31 | 24.18 | 19.70 | 8.01 | 9.97 |
| Yuyao Domestic | 46.97 | 30.45 | 24.56 | 10.30 | 11.94 |
| Yuyao Domestic | 42.75 | 29.70 | 23.78 | 10.05 | 9.53 |
| Yuyao Domestic | 40.42 | 25.87 | 26.32 | 8.63 | 10.45 |
| **Mean** | **40.03** | **26.41** | **23.84** | **9.61** | **11.07** |  | |
| **±SD** | **4.00** | **2.91** | **3.87** | **1.09** | **1.41** |  | |
| **Number** | **65** | **65** | **65** | **65** | **65** |  | |
| **Sample** | **Length (mm)** | **Height (mm)** | **Thickness (mm)** | **Top Diameter (mm)** | **Bottom Diameter (mm)** | **Comments** | |
| Nanhu Domestic | 43.62 | 24.58 | 28.32 | 11.93 | 12.95 | *Trapa acornis* Nakai, purchased at the market in Jiaxing, Zhejiang Province, China | |
| Nanhu Domestic | 40.93 | 25.59 | 27.91 | 11.50 | 13.87 |
| Nanhu Domestic | 40.35 | 26.96 | 26.08 | 10.48 | 12.71 |
| Nanhu Domestic | 39.33 | 22.17 | 23.38 | 9.36 | 11.55 |
| Nanhu Domestic | 36.33 | 20.91 | 23.74 | 8.61 | 7.37 |
| Nanhu Domestic | 34.05 | 21.34 | 14.94 | 8.77 | 7.11 |
| Nanhu Domestic | 34.69 | 20.96 | 20.14 | 8.48 | 8.70 |
| Nanhu Domestic | 41.91 | 28.04 | 27.51 | 9.39 | 11.73 |
| Nanhu Domestic | 47.77 | 25.65 | 33.37 | 10.89 | 12.47 |
| Nanhu Domestic | 40.79 | 25.73 | 23.72 | 9.76 | 12.54 |
| Nanhu Domestic | 37.09 | 23.52 | 21.58 | 9.72 | 11.78 |
| Nanhu Domestic | 43.00 | 22.27 | 30.50 | 10.50 | 10.24 |
| Nanhu Domestic | 42.24 | 26.50 | 28.47 | 9.56 | 11.56 |
| Nanhu Domestic | 42.90 | 22.35 | 24.77 | 9.32 | 11.48 |
| Nanhu Domestic | 40.57 | 21.58 | 25.61 | 9.89 | 11.78 |
| Nanhu Domestic | 42.35 | 27.85 | 21.29 | 11.59 | 12.57 |
| Nanhu Domestic | 42.54 | 26.25 | 27.65 | 9.03 | 12.06 |
| Nanhu Domestic | 38.87 | 23.78 | 19.68 | 8.98 | 11.66 |
| Nanhu Domestic | 36.74 | 21.60 | 19.82 | 8.79 | 11.45 |
| Nanhu Domestic | 39.04 | 25.33 | 20.09 | 8.94 | 11.82 |
| Nanhu Domestic | 34.57 | 23.31 | 20.06 | 10.44 | 12.00 |
| Nanhu Domestic | 41.97 | 22.82 | 23.04 | 10.87 | 11.00 |
| Nanhu Domestic | 42.30 | 25.29 | 25.55 | 8.69 | 11.98 |
| Nanhu Domestic | 48.47 | 22.92 | 32.31 | 9.35 | 11.19 |
| Nanhu Domestic | 39.10 | 27.20 | 23.55 | 10.90 | 11.68 |
| Nanhu Domestic | 36.87 | 22.28 | 19.33 | 8.23 | 10.54 |
| Nanhu Domestic | 40.36 | 28.27 | 26.40 | 10.80 | 11.34 |
| Nanhu Domestic | 36.36 | 19.66 | 20.22 | 9.52 | 11.66 |
| Nanhu Domestic | 39.16 | 28.66 | 23.11 | 10.25 | 11.54 |
| Nanhu Domestic | 34.29 | 20.37 | 20.43 | 9.30 | 10.90 |
| **Mean** | **39.95** | **24.12** | **24.09** | **9.79** | **11.37** |  | |
| **±SD** | **3.62** | **2.61** | **4.24** | **1.00** | **1.44** |  | |
| **Number** | **30** | **30** | **30** | **30** | **30** |  | |
| **Sample** | **Length (mm)** | **Height (mm)** | **Thickness (mm)** | **Top Diameter (mm)** | **Bottom Diameter (mm)** | **Comments** | |
| Yuhang Domestic | 35.86 | 21.32 | 15.98 | 6.65 | 8.91 | *Trapa bispinosa* Roxb., purchased at the market in Hangzhou, Zhejiang Province, China | |
| Yuhang Domestic | 37.43 | 17.20 | 14.98 | 7.79 | 8.58 |
| Yuhang Domestic | 35.97 | 23.24 | 16.91 | 7.65 | 8.46 |
| Yuhang Domestic | 34.65 | 23.12 | 19.46 | 6.35 | 9.97 |
| Yuhang Domestic | 36.63 | 23.38 | 22.63 | 9.39 | 10.42 |
| Yuhang Domestic | 38.30 | 21.82 | 19.38 | 7.06 | 10.18 |
| Yuhang Domestic | 35.04 | 21.48 | 18.13 | 6.54 | 10.68 |
| Yuhang Domestic | 44.04 | 23.70 | 21.38 | 9.04 | 11.57 |
| Yuhang Domestic | 39.54 | 21.72 | 17.25 | 6.77 | 8.88 |
| Yuhang Domestic | 34.90 | 20.90 | 17.04 | 5.26 | 10.13 |
| **Mean** | **37.24** | **21.79** | **18.31** | **7.25** | **9.78** |  | |
| **±SD** | **2.86** | **1.89** | **2.40** | **1.25** | **1.03** |  | |
| **Number** | **10** | **10** | **10** | **10** | **10** |  | |
| **Sample** | **Length (mm)** | **Height (mm)** | **Thickness (mm)** | **Top Diameter (mm)** | **Bottom Diameter (mm)** | **Comments** | |
| Red 2 Horns Domestic | 55.33 | 27.73 | 18.65 | 9.01 | 13.99 | *Trapa bispinosa* Roxb., purchased at the market in Jiaxing, Zhejiang Province, China | |
| Red 2 Horns Domestic | 64.10 | 26.75 | 19.57 | 7.46 | 15.39 |
| Red 2 Horns Domestic | 56.45 | 26.06 | 18.56 | 8.18 | 16.27 |
| Red 2 Horns Domestic | 63.16 | 27.47 | 19.56 | 7.84 | 16.99 |
| Red 2 Horns Domestic | 59.94 | 30.70 | 22.47 | 8.25 | 18.05 |
| Red 2 Horns Domestic | 63.59 | 28.28 | 21.45 | 7.90 | 17.75 |
| Red 2 Horns Domestic | 64.67 | 26.73 | 20.33 | 6.90 | 16.92 |
| Red 2 Horns Domestic | 59.46 | 26.03 | 16.87 | 8.65 | 13.12 |
| Red 2 Horns Domestic | 54.89 | 25.68 | 19.46 | 8.81 | 16.64 |
| Red 2 Horns Domestic | 53.48 | 25.89 | 19.01 | 6.74 | 14.12 |
| Red 2 Horns Domestic | 61.21 | 30.55 | 23.01 | 8.61 | 17.40 |
| Red 2 Horns Domestic | 62.83 | 28.43 | 22.47 | 9.15 | 18.60 |
| Red 2 Horns Domestic | 63.92 | 29.26 | 23.46 | 8.91 | 19.25 |
| Red 2 Horns Domestic | 61.08 | 31.08 | 21.25 | 8.48 | 16.14 |
| Red 2 Horns Domestic | 65.60 | 29.13 | 22.13 | 7.79 | 15.19 |
| Red 2 Horns Domestic | 58.38 | 25.99 | 18.82 | 6.40 | 14.08 |
| Red 2 Horns Domestic | 61.34 | 27.21 | 20.05 | 8.92 | 15.54 |
| Red 2 Horns Domestic | 60.58 | 24.59 | 21.66 | 8.27 | 14.60 |
| Red 2 Horns Domestic | 66.17 | 27.09 | 24.50 | 7.30 | 18.45 |
| Red 2 Horns Domestic | 58.92 | 24.40 | 19.41 | 8.64 | 15.40 |
| Red 2 Horns Domestic | 61.08 | 28.45 | 22.19 | 9.12 | 16.39 |
| Red 2 Horns Domestic | 54.80 | 25.55 | 21.59 | 6.61 | 14.88 |
| Red 2 Horns Domestic | 53.79 | 26.95 | 18.35 | 6.84 | 13.19 |
| Red 2 Horns Domestic | 66.01 | 28.84 | 19.35 | 7.24 | 16.80 |
| Red 2 Horns Domestic | 64.77 | 28.83 | 20.14 | 8.73 | 13.15 |
| Red 2 Horns Domestic | 63.86 | 29.52 | 19.19 | 6.54 | 15.57 |
| Red 2 Horns Domestic | 54.94 | 26.12 | 21.00 | 7.59 | 16.73 |
| Red 2 Horns Domestic | 60.26 | 26.36 | 18.89 | 7.82 | 13.69 |
| Red 2 Horns Domestic | 59.98 | 26.19 | 19.48 | 7.35 | 14.12 |
| Red 2 Horns Domestic | 64.99 | 26.74 | 20.58 | 9.44 | 15.22 |
| Red 2 Horns Domestic | 63.74 | 28.54 | 19.71 | 8.73 | 15.58 |
| Red 2 Horns Domestic | 59.43 | 28.35 | 19.85 | 7.33 | 14.18 |
| Red 2 Horns Domestic | 62.12 | 29.86 | 23.53 | 8.25 | 17.59 |
| Red 2 Horns Domestic | 59.55 | 26.78 | 17.78 | 7.43 | 15.47 |
| Red 2 Horns Domestic | 61.28 | 26.96 | 20.45 | 7.53 | 15.94 |
| Red 2 Horns Domestic | 57.00 | 27.02 | 18.24 | 7.36 | 16.39 |
| Red 2 Horns Domestic | 60.08 | 28.79 | 19.33 | 7.15 | 16.87 |
| **Mean** | **60.62** | **27.54** | **20.33** | **7.93** | **15.83** |  | |
| **±SD** | **3.62** | **1.66** | **1.77** | **0.84** | **1.60** |  | |
| **Number** | **37** | **37** | **37** | **37** | **37** |  | |
| Red 4 Horns Domestic | 77.00 | 32.32 | 27.16 | 12.17 | 15.66 | *Trapa bispinosa* Roxb., purchased at the market in Jiaxing, Zhejiang Province, China | |
| Red 4 Horns Domestic | 73.08 | 30.95 | 24.85 | 12.61 | 15.50 |
| Red 4 Horns Domestic | 71.03 | 30.45 | 24.19 | 13.64 | 14.50 |
| Red 4 Horns Domestic | 70.72 | 27.89 | 22.61 | 10.82 | 14.33 |
| Red 4 Horns Domestic | 69.93 | 29.13 | 22.39 | 10.51 | 15.88 |
| Red 4 Horns Domestic | 53.42 | 28.76 | 29.16 | 12.42 | 12.66 |
| Red 4 Horns Domestic | 56.08 | 26.82 | 25.46 | 7.86 | 13.39 |
| Red 4 Horns Domestic | 60.98 | 28.14 | 23.05 | 11.60 | 14.31 |
| Red 4 Horns Domestic | 66.08 | 25.13 | 22.71 | 10.07 | 12.92 |
| Red 4 Horns Domestic | 60.15 | 29.57 | 26.25 | 8.31 | 14.03 |
| Red 4 Horns Domestic | 54.15 | 31.42 | 26.33 | 9.58 | 14.89 |
| Red 4 Horns Domestic | 67.88 | 28.13 | 24.36 | 10.50 | 14.73 |
| Red 4 Horns Domestic | 72.56 | 28.60 | 26.73 | 8.59 | 15.34 |
| Red 4 Horns Domestic | 53.82 | 29.99 | 26.94 | 8.53 | 12.43 |
| Red 4 Horns Domestic | 64.70 | 31.63 | 26.79 | 8.70 | 15.47 |
| Red 4 Horns Domestic | 68.18 | 29.99 | 22.89 | 11.09 | 16.97 |
| Red 4 Horns Domestic | 55.39 | 26.18 | 26.45 | 11.27 | 14.23 |
| Red 4 Horns Domestic | 61.75 | 27.09 | 22.50 | 10.57 | 16.56 |
| Red 4 Horns Domestic | 66.20 | 27.80 | 22.79 | 10.25 | 18.97 |
| Red 4 Horns Domestic | 58.69 | 27.63 | 23.49 | 9.11 | 14.18 | loss of one shoulder horn | |
| Red 4 Horns Domestic | 65.64 | 33.92 | 27.26 | 12.76 | 15.28 |  | |
| Red 4 Horns Domestic | 68.82 | 30.77 | 24.61 | 10.48 | 18.04 |
| Red 4 Horns Domestic | 68.93 | 32.80 | 27.88 | 10.47 | 16.48 |
| Red 4 Horns Domestic | 60.14 | 27.63 | 20.79 | 7.61 | 13.41 |
| Red 4 Horns Domestic | 55.61 | 26.99 | 25.55 | 8.31 | 13.99 | little vitiation of two shoulder horns | |
| Red 4 Horns Domestic | 65.98 | 28.48 | 24.67 | 7.51 | 15.97 |  | |
| Red 4 Horns Domestic | 70.48 | 29.48 | 25.69 | 10.88 | 14.89 |
| Red 4 Horns Domestic | 67.18 | 30.30 | 22.43 | 9.19 | 16.68 |
| Red 4 Horns Domestic | 73.46 | 25.87 | 25.63 | 9.04 | 13.80 |
| Red 4 Horns Domestic | 60.94 | 29.93 | 30.00 | 8.99 | *Not determined* | one bent shoulder horn | |
| Red 4 Horns Domestic | 66.93 | 29.12 | 25.14 | 11.16 | 14.27 |  | |
| Red 4 Horns Domestic | 67.74 | 25.87 | 22.02 | 9.03 | 12.80 |
| Red 4 Horns Domestic | 52.86 | 30.25 | 27.40 | 7.84 | 13.32 |
| Red 4 Horns Domestic | 55.12 | 28.32 | 25.19 | 8.24 | 14.07 |
| Red 4 Horns Domestic | 67.89 | 30.77 | 22.48 | 9.25 | 18.13 |
| Red 4 Horns Domestic | 56.61 | 33.87 | 24.41 | 7.85 | 13.55 |
| Red 4 Horns Domestic | 69.34 | 32.95 | 27.63 | 10.48 | 15.11 |
| **Mean** | **64.20** | **29.32** | **25.02** | **9.93** | **14.91** |  | |
| **±SD** | **6.70** | **2.24** | **2.18** | **1.61** | **1.58** |  | |
| **Number** | **37** | **37** | **37** | **37** | **36** |  | |

Supplementary Table 2 Archaeological Samples

| **Sample** | **Length (mm)** | **Height (mm)** | **Thickness (mm)** | **Top Diameter (mm)** | **Bottom Diameter (mm)** | **Comments** | **Type** |
| --- | --- | --- | --- | --- | --- | --- | --- |
| T104⑧ Preserved | 26.16 | 18.24 | *Not determined* | 6.68 | 5.16 | little vitiation one horn | *Trapa bispinosa* Roxb., collected from the Tianluoshan site |
| T104⑧ Preserved | 18.91 | 17.45 | *Not determined* | 6.49 | 6.57 | vitiation, the body is deformed |
| T104⑧ Preserved | 25.95 | 14.08 | *Not determined* | 5.68 | 5.05 | little vitiation |
| T104⑧ Preserved | 22.57 | 14.97 | *Not determined* | 5.06 | 4.94 | little vitiation, pressed |
| T104⑧ Preserved | 26.87 | 13.25 | *Not determined* | 5.10 | 5.26 | little vitiation, pressed |
| T104⑧ Preserved | 25.72 | *Not determined* | *Not determined* | 6.14 | 4.02 | little vitiation, pressed |
| T104⑧ Preserved | 27.27 | 14.66 | *Not determined* | 5.39 | 4.75 | little vitiation, pressed |
| T104⑧ Preserved | 28.13 | 13.36 | *Not determined* | 6.22 | *Not determined* | little vitiation, pressed |
| **Mean** | **25.20** | **15.14** |  | **5.85** | **5.11** |  |  |
| **±SD** | **3.02** | **1.96** |  | **0.63** | **0.76** |  |  |
| **Number** | **8** | **7** |  | **8** | **7** |  |  |
| **Sample** | **Length (mm)** | **Height (mm)** | **Thickness (mm)** | **Top Diameter (mm)** | **Bottom Diameter (mm)** | **Comments** | **Type** |
| T206⑧ Preserved | 30.12 | 12.79 | 10.57 | 6.11 | 9.83 | little vitiation of both horns | *Trapa bispinosa* Roxb., collected from the Tianluoshan site |
| T206⑧ Preserved | 34.48 | 14.67 | 14.21 | 5.96 | 12.32 |  |
| T206⑧ Preserved | 28.80 | 16.02 | 8.71 | 5.49 | 11.58 |  |
| T206⑧ Preserved | 29.87 | 12.60 | 12.66 | 4.97 | 10.74 |  |
| T206⑧ Preserved | 34.73 | 14.00 | 18.82 | 6.08 | 12.85 |  |
| T206⑧ Preserved | 29.45 | 13.80 | 13.37 | 5.08 | 9.52 |  |
| T206⑧ Preserved | 29.48 | 15.71 | 10.03 | 5.99 | 11.47 |  |
| T206⑧ Preserved | 28.34 | 17.50 | 10.47 | 6.76 | 11.35 | little vitiation of one horn |
| T206⑧ Preserved | 28.86 | 18.79 | 8.41 | 5.35 | 11.23 |  |
| T206⑧ Preserved | 30.19 | 15.22 | 11.52 | 5.66 | 11.47 |  |
| T206⑧ Preserved | 29.16 | 13.20 | 12.15 | 4.98 | 12.02 | little vitiation of one horn |
| T206⑧ Preserved | 31.74 | 12.11 | 15.68 | 5.24 | 9.63 | little vitiation of one horn |
| T206⑧ Preserved | 33.51 | 17.25 | 13.71 | 5.91 | 11.72 |  |
| T206⑧ Preserved | 31.63 | 14.53 | 14.54 | 5.77 | 11.62 |  |
| T206⑧ Preserved | 29.27 | 17.25 | 15.37 | 4.94 | *Not determined* |  |
| T206⑧ Preserved | 32.28 | 16.71 | 9.35 | 5.46 | 10.63 |  |
| T206⑧ Preserved | 24.95 | 16.69 | 11.49 | 6.74 | 7.26 | little vitiation of one horn |
| T206⑧ Preserved | 28.27 | 16.44 | 10.82 | 6.16 | 13.38 |  |
| T206⑧ Preserved | 28.43 | 13.70 | 11.85 | 5.63 | 9.05 |  |
| T206⑧ Preserved | 29.13 | 17.78 | 14.24 | 5.90 | 15.15 |  |
| T206⑧ Preserved | 28.84 | 18.14 | 9.55 | 7.59 | 4.73 |  |
| T206⑧ Preserved | 26.01 | 9.05 | 13.13 | 4.54 | *Not determined* |  |
| T206⑧ Preserved | 37.24 | 14.40 | 9.48 | 4.65 | 8.98 |  |
| T206⑧ Preserved | 27.21 | 15.67 | 12.72 | 5.96 | 12.84 | little vitiation of one horn |
| T206⑧ Preserved | 19.12 | 18.56 | 10.72 | 6.00 | 7.98 |  |
| T206⑧ Preserved | 33.34 | 19.46 | 7.15 | 6.79 | *Not determined* |  |
| T206⑧ Preserved | 22.22 | 13.29 | 12.44 | 4.76 | 8.20 | little vitiation of one horn |
| T206⑧ Preserved | 30.97 | 18.80 | 11.06 | 5.20 | 10.29 |  |
| T206⑧ Preserved | 31.16 | 16.67 | 14.30 | 5.83 | 12.54 | little vitiation of one horn |
| T206⑧ Preserved | 31.36 | 13.33 | 12.11 | 5.21 | 9.60 |  |
| T206⑧ Preserved | 29.82 | 15.31 | 9.17 | 5.22 | 9.20 |  |
| T206⑧ Preserved | 32.01 | 17.82 | *Not determined* | 5.32 | 9.68 |  |
| T206⑧ Preserved | 34.11 | 17.01 | 10.06 | 5.73 | 12.48 |  |
| T206⑧ Preserved | 21.26 | 13.48 | 8.87 | 5.00 | 6.49 | little vitiation of one horn |
| T206⑧ Preserved | 25.72 | 14.41 | 7.70 | 6.26 | 4.85 | little vitiation of one horn |
| T206⑧ Preserved | 30.32 | 16.06 | 13.89 | 6.02 | 10.71 |  |
| T206⑧ Preserved | 28.50 | 13.50 | 13.79 | 4.46 | 7.52 |  |
| T206⑧ Preserved | 24.22 | 10.82 | 9.30 | 4.11 | 6.17 |  |
| **Mean** | **29.37** | **15.33** | **11.71** | **5.60** | **10.15** |  |  |
| **±SD** | **3.72** | **2.37** | **2.53** | **0.72** | **2.41** |  |  |
| **Number** | **38** | **38** | **37** | **38** | **35** |  |  |
| **Layer 8 summary information for the water chestnuts from Tianluoshan** | | | | | | | |
| **Layer 8 Mean** | **28.65** | **15.30** | **11.71** | **5.64** | **9.31** |  |  |
| **Layer 8 ±SD** | **3.92** | **2.29** | **2.53** | **0.70** | **2.92** |  |  |
| **Layer 8 Number** | **46** | **45** | **37** | **46** | **42** |  |  |
| **Sample** | **Length (mm)** | **Height (mm)** | **Thickness (mm)** | **Top Diameter (mm)** | **Bottom Diameter (mm)** | **Comments** | **Type** |
| T205⑦ Waterlogged | 28.49 | 16.09 | 12.32 | 4.27 | 10.04 | one horn deformed | *Trapa bispinosa* Roxb., collected from the Tianluoshan site |
| T205⑦ Waterlogged | 28.42 | 15.98 | 15.63 | 3.44 | 9.76 |  |
| T205⑦ Waterlogged | 27.80 | 19.11 | 10.57 | 7.17 | 7.61 |  |
| T205⑦ Waterlogged | 31.07 | 20.27 | 11.24 | 5.96 | 12.98 |  |
| T205⑦ Waterlogged | 28.94 | 18.12 | 5.81 | 6.06 | 11.53 | pressed |
| T205⑦ Waterlogged | 27.13 | 15.78 | 14.44 | 5.58 | 10.76 |  |
| T205⑦ Waterlogged | 27.04 | 14.78 | 15.47 | 4.66 | 8.08 | little vitiation of one horn |
| T205⑦ Waterlogged | 31.31 | 17.86 | 10.87 | 6.18 | 11.83 | little vitiation of one horn |
| T205⑦ Waterlogged | 26.42 | 16.62 | 11.44 | 4.95 | 9.65 |  |
| T205⑦ Waterlogged | 31.70 | 12.68 | 16.32 | 5.71 | 9.44 |  |
| T205⑦ Waterlogged | 23.77 | 14.56 | 8.50 | 4.64 | 6.59 |  |
| T205⑦ Waterlogged | 39.87 | 21.78 | 10.27 | 7.21 | 14.81 |  |
| T205⑦ Waterlogged | 22.45 | 9.51 | 6.88 | 4.10 | 4.23 | four horns |
| T205⑦ Waterlogged | 20.26 | 9.60 | 9.11 | 3.72 | 4.36 | four horns |
| T205⑦ Waterlogged | 22.89 | 10.91 | 9.38 | 4.39 | 6.26 |  |
| T205⑦ Waterlogged | 21.56 | 12.93 | 7.95 | 5.04 | 4.40 |  |
| T205⑦ Waterlogged | 33.61 | 17.16 | 12.91 | 9.68 | 9.03 |  |
| T205⑦ Waterlogged | 22.90 | 14.18 | 13.54 | 6.83 | 9.84 | vitiation of two horns |
| T205⑦ Waterlogged | 33.87 | 18.10 | 5.82 | 6.24 | 9.63 | pressed, vitiation of one horn |
| T205⑦ Waterlogged | 29.10 | 16.49 | 9.25 | 6.37 | 9.51 | pressed |
| T205⑦ Waterlogged | 34.59 | 20.20 | 7.94 | 5.91 | 25.82 | pressed |
| T205⑦ Waterlogged | 29.38 | 17.51 | 10.67 | 5.61 | 9.64 |  |
| T205⑦ Waterlogged | 23.33 | 12.46 | 12.06 | 5.93 | 7.54 | vitiation of one horn |
| T205⑦ Waterlogged | 41.52 | 18.28 | 12.98 | 8.12 | 7.91 |  |
| T205⑦ Waterlogged | 34.48 | 19.87 | 8.81 | 6.11 | 9.07 |  |
| T205⑦ Waterlogged | 30.48 | 13.58 | 9.66 | 6.47 | 6.23 | partly vitiation of base |
| T205⑦ Waterlogged | 36.33 | 19.20 | 10.13 | 8.07 | 12.64 |  |
| T205⑦ Waterlogged | 34.86 | 18.19 | 15.56 | 6.90 | 11.53 | little vitiation of two horns |
| T205⑦ Waterlogged | 24.15 | 14.93 | 9.24 | 7.72 | 6.42 |  |
| T205⑦ Waterlogged | 34.22 | 16.38 | 8.72 | 7.51 | 8.07 |  |
| T205⑦ Waterlogged | 32.92 | 16.00 | 15.64 | 5.77 | 11.40 |  |
| T205⑦ Waterlogged | 33.34 | 11.85 | 13.97 | 6.43 | 6.66 |  |
| T205⑦ Waterlogged | 31.85 | 21.16 | 18.13 | 4.98 | 10.97 | little vitiation of one horn |
| T205⑦ Waterlogged | 21.18 | 21.48 | 12.50 | 6.20 | 9.30 |  |
| T205⑦ Waterlogged | 35.80 | 14.90 | 10.54 | 4.90 | 8.89 |  |
| T205⑦ Waterlogged | 27.30 | 11.62 | 12.27 | 4.60 | 9.13 |  |
| T205⑦ Waterlogged | 25.10 | 18.30 | 13.78 | 3.59 | 10.27 | partly vitiation of base |
| T205⑦ Waterlogged | 34.38 | 18.99 | 7.93 | 6.58 | 13.82 | pressed |
| T205⑦ Waterlogged | 31.30 | 17.78 | 8.82 | 6.77 | 8.39 | pressed, vitiation of one horn |
| T205⑦ Waterlogged | 24.82 | 17.37 | 4.86 | 6.16 | 5.67 | pressed, vitiation of one horn |
| T205⑦ Waterlogged | 32.70 | 19.42 | 9.17 | 5.67 | 13.20 | pressed, little vitiation of one horn and base |
| T205⑦ Waterlogged | 31.42 | 14.00 | 12.08 | 5.85 | 10.38 | little vitiation of two horns |
| T205⑦ Waterlogged | 32.33 | 15.96 | 13.71 | 6.44 | 12.17 |  |
| T205⑦ Waterlogged | 27.75 | 15.52 | 10.94 | 4.80 | 11.22 |  |
| T205⑦ Waterlogged | 27.91 | 14.94 | 9.69 | 7.62 | 6.81 |  |
| T205⑦ Waterlogged | 32.01 | 15.42 | 13.63 | 5.12 | 12.31 |  |
| T205⑦ Waterlogged | 34.70 | 14.19 | 15.66 | 6.10 | 12.04 | deformed |
| T205⑦ Waterlogged | 30.58 | 19.50 | 12.24 | 5.91 | 11.78 | vitiation of one horn |
| T205⑦ Waterlogged | 32.40 | 17.92 | 16.35 | 7.38 | 13.26 | vitiation of one horn |
| T205⑦ Waterlogged | 35.18 | 19.33 | 14.15 | 5.73 | 12.73 |  |
| T205⑦ Waterlogged | 28.84 | 14.99 | 10.61 | 5.99 | 6.02 | little vitiation of one horn |
| T205⑦ Waterlogged | 30.49 | 14.17 | 15.82 | 4.29 | 11.04 | deformed |
| T205⑦ Waterlogged | 34.07 | 12.08 | 10.31 | 6.40 | 7.90 |  |
| T205⑦ Waterlogged | 36.55 | 19.27 | 11.19 | 6.78 | 10.57 | pressed |
| T205⑦ Waterlogged | 32.72 | 14.58 | 14.58 | 4.95 | 10.35 |  |
| T205⑦ Waterlogged | 31.42 | 18.09 | 9.23 | 5.62 | 11.46 | pressed, little vitiation of one horn |
| T205⑦ Waterlogged | 32.60 | 17.84 | 12.52 | 6.30 | 13.63 |  |
| T205⑦ Waterlogged | 36.79 | 17.29 | 15.19 | 6.60 | 13.16 |  |
| T205⑦ Waterlogged | 30.23 | 17.87 | 7.92 | 7.28 | 8.01 | pressed, little vitiation of one horn |
| T205⑦ Waterlogged | 28.68 | 13.61 | 14.96 | 5.28 | 11.89 |  |
| T205⑦ Waterlogged | 31.72 | 12.85 | 15.42 | 6.36 | 9.20 | deformed |
| T205⑦ Waterlogged | 29.88 | 12.93 | *Not determined* | 6.08 | 6.33 | deformed, pressed, little vitiation of one horn |
| T205⑦ Waterlogged | 30.89 | 17.79 | 9.32 | 6.59 | 10.02 | pressed |
| T205⑦ Waterlogged | 28.89 | 17.64 | 9.54 | 7.72 | 6.68 | little vitiation of one horn |
| T205⑦ Waterlogged | 32.09 | 13.55 | 7.67 | 5.30 | 6.08 |  |
| T205⑦ Waterlogged | 34.11 | 14.77 | 12.00 | 5.96 | 11.79 |  |
| T205⑦ Waterlogged | 26.09 | 14.12 | 11.80 | 6.09 | 9.25 |  |
| T205⑦ Waterlogged | 20.20 | 9.57 | 9.44 | 4.72 | 4.83 | little vitiation of one horn |
| T205⑦ Waterlogged | 36.43 | 18.49 | 6.70 | 8.59 | 9.30 | little vitiation of one horn, pressed |
| T205⑦ Waterlogged | 32.55 | 14.37 | 14.24 | 6.00 | 11.39 |  |
| T205⑦ Waterlogged | 28.18 | 13.96 | 9.94 | 6.79 | 8.70 | little vitiation of two horns |
| T205⑦ Waterlogged | 32.05 | 13.53 | 13.22 | 5.22 | 6.98 |  |
| T205⑦ Waterlogged | 27.57 | 17.93 | 10.12 | 7.60 | 7.97 | little vitiation of one horn, pressed |
| **Mean** | **30.27** | **16.03** | **11.41** | **6.02** | **9.70** |  |  |
| **±SD** | **4.54** | **2.89** | **2.95** | **1.19** | **3.16** |  |  |
| **Number** | **73** | **73** | **72** | **73** | **73** |  |  |
| **Sample** | **Length (mm)** | **Height (mm)** | **Thickness (mm)** | **Top Diameter (mm)** | **Bottom Diameter (mm)** | **Comments** | **Type** |
| T305⑦ Waterlogged | 29.91 | 16.69 | 11.15 | 6.07 | 11.29 | little vitiation of both horns, pressed | *Trapa bispinosa* Roxb., collected from the Tianluoshan site |
| T305⑦ Waterlogged | 33.15 | 17.99 | 12.95 | 6.10 | 13.23 | little vitiation of both horns |
| T305⑦ Waterlogged | 23.58 | 11.96 | 4.55 | 6.20 | 4.74 | little vitiation of both horns, crooked |
| T305⑦ Waterlogged | 36.49 | 19.94 | 14.43 | 6.26 | 14.27 | little vitiation of both horns, crooked |
| T305⑦ Waterlogged | 25.57 | 11.89 | 7.81 | 7.71 | 4.66 |  |
| T305⑦ Waterlogged | 31.12 | 15.05 | 14.38 | 6.84 | 10.66 | vitiation of one horn |
| T305⑦ Waterlogged | 28.47 | 14.34 | 13.48 | 6.20 | 11.15 | little vitiation of both horns |
| T305⑦ Waterlogged | 27.71 | 15.18 | 13.64 | 5.83 | 11.38 | little vitiation of both horns and the base |
| T305⑦ Waterlogged | 30.10 | 17.77 | 13.54 | 4.80 | 11.66 | little vitiation of both horns |
| T305⑦ Waterlogged | 29.30 | 18.72 | 8.76 | 7.76 | 8.80 | little vitiation of both horns, pressed |
| T305⑦ Waterlogged | 26.88 | 14.75 | 15.56 | 3.87 | 10.94 | little vitiation of both horns |
| T305⑦ Waterlogged | 31.90 | 14.45 | 14.77 | 6.34 | 12.33 | little vitiation of both horns, crooked |
| T305⑦ Waterlogged | 27.54 | 13.15 | 9.01 | 4.97 | 10.64 | little vitiation of both horns |
| T305⑦ Waterlogged | 26.75 | 16.57 | 13.81 | 4.69 | 11.75 | little vitiation of both horns |
| T305⑦ Waterlogged | 27.84 | 15.35 | 13.03 | 6.29 | 10.18 | little vitiation of both horns, carbonized |
| T305⑦ Waterlogged | *Not determined* | 18.36 | 18.12 | 5.16 | 14.78 | little vitiation of both horns |
| T305⑦ Waterlogged | 31.36 | 11.10 | 15.52 | 6.77 | 12.78 | little vitiation of both horns,crooked |
| T305⑦ Waterlogged | 30.49 | 17.58 | 7.78 | 7.12 | 7.69 | little vitiation of both horns |
| T305⑦ Waterlogged | 34.11 | 19.05 | 10.73 | 6.28 | 14.60 | little vitiation of both horns |
| T305⑦ Waterlogged | 34.78 | 15.62 | 6.16 | 6.16 | 11.62 | little vitiation of both horns |
| T305⑦ Waterlogged | 30.74 | 15.13 | 5.88 | 5.88 | 11.23 | little vitiation of both horns |
| T305⑦ Waterlogged | 19.79 | 13.45 | 3.16 | 3.16 | 7.53 | little vitiation of both horns |
| T305⑦ Waterlogged | 24.96 | 15.71 | 5.52 | 5.52 | 7.73 |  |
| T305⑦ Waterlogged | *Not determined* | 15.18 | 3.81 | 3.81 | 11.80 |  |
| T305⑦ Waterlogged | 28.19 | 15.24 | 5.18 | 5.18 | 9.92 |  |
| T305⑦ Waterlogged | 32.91 | 16.42 | 12.83 | 6.70 | 11.40 | little vitiation of both horns |
| T305⑦ Waterlogged | 29.77 | 16.20 | 13.76 | 5.72 | *Not determined* | little vitiation of both horns and the base |
| T305⑦ Waterlogged | 30.26 | 16.32 | 12.56 | 6.14 | 12.73 | little vitiation of both horns |
| T305⑦ Waterlogged | 31.99 | 13.05 | 8.66 | 6.16 | 5.97 |  |
| T305⑦ Waterlogged | 22.66 | 12.01 | 10.68 | 4.29 | 9.12 | little vitiation of both horns |
| T305⑦ Waterlogged | 35.22 | 17.02 | 12.38 | 5.77 | 13.68 | little vitiation of both horns |
| T305⑦ Waterlogged | 24.97 | 13.69 | 11.64 | 5.14 | 10.78 | little vitiation of both horns |
| T305⑦ Waterlogged | 31.84 | 19.26 | 9.48 | 6.88 | 8.26 | little vitiation of both horns |
| T305⑦ Waterlogged | 28.15 | 11.70 | 9.13 | 6.85 | 4.70 |  |
| T305⑦ Waterlogged | 34.96 | 15.99 | 17.40 | 6.30 | 11.83 | crooked |
| T305⑦ Waterlogged | 30.03 | 14.91 | 12.83 | 4.93 | 11.29 | little vitiation of two horns |
| T305⑦ Waterlogged | 28.10 | 13.88 | 11.27 | 8.24 | 7.18 | little vitiation of two horns |
| T305⑦ Waterlogged | 36.13 | 17.57 | 13.56 | 5.25 | 13.54 |  |
| T305⑦ Waterlogged | 22.61 | 15.31 | 9.00 | 5.46 | 6.16 | little vitiation of two horns |
| T305⑦ Waterlogged | 30.75 | 17.22 | 13.23 | 4.22 | 11.17 | little vitiation of two horns |
| T305⑦ Waterlogged | 31.05 | 14.57 | 13.87 | 5.06 | 12.45 | little vitiation of two horns |
| T305⑦ Waterlogged | 34.33 | 15.65 | 12.63 | 5.66 | 11.96 |  |
| T305⑦ Waterlogged | 37.90 | 18.65 | 6.23 | 5.68 | 7.08 | pressed |
| T305⑦ Waterlogged | 34.46 | 19.41 | 11.68 | 5.80 | 13.56 |  |
| T305⑦ Waterlogged | 32.56 | 13.79 | 15.55 | 5.53 | 13.15 | little vitiation of two horns |
| T305⑦ Waterlogged | 28.76 | 14.98 | 8.40 | 5.93 | 6.67 |  |
| T305⑦ Waterlogged | 34.78 | 14.79 | 13.80 | 6.66 | 13.82 | little vitiation of two horns |
| T305⑦ Waterlogged | 29.96 | 15.69 | 14.04 | 5.43 | 12.27 | little vitiation of two horns |
| T305⑦ Waterlogged | 27.38 | 16.47 | 10.09 | 6.77 | 7.65 | little vitiation of two horns, pressed |
| T305⑦ Waterlogged | 30.02 | 15.96 | 11.68 | 6.29 | 11.84 | little vitiation of two horns |
| T305⑦ Waterlogged | 21.75 | 11.21 | 8.24 | 5.29 | 4.53 | little vitiation of two horns, pressed, crooked |
| T305⑦ Waterlogged | 28.25 | 17.67 | *Not determined* | 5.34 | 10.24 | little vitiation of two horns, pressed, crooked |
| **Mean** | **29.85** | **15.57** | **11.12** | **5.82** | **10.40** |  |  |
| **±SD** | **4.01** | **2.20** | **3.57** | **0.99** | **2.81** |  |  |
| **Number** | **50** | **52** | **51** | **52** | **51** |  |  |
| **Layer 7 summary information for the water chestnuts from Tianluoshan** | | | | | | | |
| **Layer 7 Mean** | **30.10** | **15.84** | **11.29** | **5.94** | **9.99** |  |  |
| **Layer 7 ±SD** | **4.32** | **2.62** | **3.21** | **1.11** | **3.03** |  |  |
| **Layer 7 Number** | **123** | **125** | **123** | **125** | **124** |  |  |
| **Sample** | **Length (mm)** | **Height (mm)** | **Thickness (mm)** | **Top Diameter (mm)** | **Bottom Diameter (mm)** | **Comments** | **Type** |
| H69⑥ Waterlogged | 29.90 | 15.18 | 13.85 | 6.35 | 11.22 | little vitiation of both horns | *Trapa bispinosa* Roxb., collected from the Tianluoshan site |
| H69⑥ Waterlogged | 37.93 | 17.37 | 18.90 | 8.02 | 12.20 | little vitiation of both horns |
| H69⑥ Waterlogged | 34.28 | 18.83 | 15.08 | 5.48 | 13.54 | little vitiation of both horns |
| H69⑥ Waterlogged | 30.70 | 15.24 | 13.60 | 6.03 | 10.45 | little vitiation of both horns |
| H69⑥ Waterlogged | 35.48 | 10.91 | 17.68 | 6.37 | 10.06 | crooked |
| H69⑥ Waterlogged | 32.65 | 18.56 | 15.19 | 5.40 | 12.59 | little vitiation of both horns |
| H69⑥ Waterlogged | 31.11 | 18.64 | 13.25 | 6.69 | 13.69 | little vitiation of both horns, crooked |
| H69⑥ Waterlogged | 34.93 | 19.23 | 12.11 | 6.86 | 12.32 | little vitiation of both horns |
| H69⑥ Waterlogged | 32.01 | 13.00 | 17.90 | 6.05 | 10.68 | short |
| H69⑥ Waterlogged | 34.71 | 18.05 | 14.72 | 5.98 | 11.50 |  |
| H69⑥ Waterlogged | 31.78 | 15.92 | 14.28 | 7.21 | 12.58 |  |
| H69⑥ Waterlogged | 31.94 | 19.98 | 9.15 | 5.01 | 11.43 | little vitiation of both horns, pressed |
| H69⑥ Waterlogged | 30.98 | 16.64 | 15.50 | 5.10 | 11.41 | little vitiation of both horns |
| H69⑥ Waterlogged | 30.67 | 17.25 | 16.08 | 4.63 | 11.50 | little vitiation of both horns |
| H69⑥ Waterlogged | 34.24 | 18.15 | 17.33 | 7.57 | 13.78 | little vitiation of both horns |
| H69⑥ Waterlogged | 33.13 | 17.53 | 15.33 | 5.92 | 13.44 | little vitiation of both horns |
| H69⑥ Waterlogged | 38.87 | 19.50 | 11.05 | 6.59 | 13.58 | little vitiation of both horns |
| H69⑥ Waterlogged | 32.56 | 17.62 | 14.83 | 5.90 | 12.05 | little vitiation of both horns |
| H69⑥ Waterlogged | 33.67 | 19.23 | 15.73 | 6.94 | 14.95 | little vitiation of both horns |
| H69⑥ Waterlogged | 33.99 | 17.29 | 15.83 | 5.88 | 13.24 | little vitiation of both horns, big crown |
| H69⑥ Waterlogged | 26.50 | 16.40 | 10.77 | 7.73 | 9.34 | little vitiation of both horns |
| H69⑥ Waterlogged | 28.12 | 16.46 | 8.18 | 7.29 | 6.99 |  |
| H69⑥ Waterlogged | 30.89 | 17.86 | 13.85 | 5.84 | 10.38 | little vitiation of both horns |
| H69⑥ Waterlogged | 35.13 | 13.75 | 15.72 | 5.12 | 10.42 | little vitiation of both horns, short, crooked |
| H69⑥ Waterlogged | 32.75 | 15.61 | 15.77 | 6.06 | 12.63 | little vitiation of both horns |
| H69⑥ Waterlogged | 34.81 | 18.81 | 9.45 | 7.09 | 7.54 | little vitiation of both horns |
| H69⑥ Waterlogged | 24.24 | 19.45 | 9.94 | 7.51 | 7.72 | little vitiation of both horns |
| H69⑥ Waterlogged | 36.06 | 19.67 | 11.23 | 6.87 | 13.10 | little vitiation of both horns, crooked, pressed |
| H69⑥ Waterlogged | 33.09 | 18.88 | 17.38 | 6.76 | 13.01 | little vitiation of both horns |
| H69⑥ Waterlogged | 33.25 | 16.69 | 11.24 | 5.71 | 13.80 | little vitiation of both horns |
| H69⑥ Waterlogged | 32.69 | 14.63 | 14.56 | 7.05 | 11.73 | little vitiation of both horns |
| H69⑥ Waterlogged | 37.41 | 22.36 | 10.09 | 7.34 | 14.24 | little vitiation of both horns, pressed |
| H69⑥ Waterlogged | 33.56 | 15.70 | 11.52 | 8.40 | 7.23 | little vitiation of both horns |
| H69⑥ Waterlogged | 34.37 | 16.83 | 15.83 | 7.02 | 11.73 | little vitiation of both horns |
| H69⑥ Waterlogged | 34.07 | 15.86 | 15.88 | 5.79 | 11.23 | little vitiation of both horns |
| H69⑥ Waterlogged | 34.40 | 16.36 | 15.58 | 6.19 | 12.31 |  |
| H69⑥ Waterlogged | 32.71 | 17.81 | 14.61 | 7.25 | 13.29 | little vitiation of both horns |
| H69⑥ Waterlogged | 34.13 | 17.93 | 13.07 | 7.10 | 12.79 |  |
| H69⑥ Waterlogged | 30.24 | 18.93 | 16.16 | 4.92 | 14.22 | little vitiation of both horns |
| H69⑥ Waterlogged | 32.75 | 16.77 | 15.49 | 7.74 | 13.35 | little vitiation of both horns |
| H69⑥ Waterlogged | 31.47 | 17.70 | 14.43 | 5.83 | 10.89 | little vitiation of both horns |
| H69⑥ Waterlogged | 34.00 | 16.41 | 11.47 | 5.43 | 9.73 | little vitiation of both horns |
| H69⑥ Waterlogged | 33.11 | 16.98 | 14.24 | 6.33 | 10.81 | little vitiation of both horns |
| H69⑥ Waterlogged | 35.94 | 17.48 | 15.45 | 7.28 | 13.13 | little vitiation of both horns |
| H69⑥ Waterlogged | 32.32 | 16.65 | 16.18 | 6.08 | 12.45 | little vitiation of both horns |
| H69⑥ Waterlogged | 33.45 | 16.93 | 15.77 | 5.93 | 11.80 | little vitiation of both horns |
| H69⑥ Waterlogged | 26.69 | 16.87 | 8.66 | 6.95 | 7.85 | little vitiation of both horns, big crown |
| H69⑥ Waterlogged | 30.62 | 17.86 | 15.06 | 7.38 | 12.55 | little vitiation of both horns |
| H69⑥ Waterlogged | 32.52 | 18.92 | 15.67 | 5.71 | 11.34 | little vitiation of both horns, crooked |
| H69⑥ Waterlogged | 30.87 | 12.89 | 16.19 | 4.89 | 10.64 | little vitiation of both horns, crooked |
| H69⑥ Waterlogged | 32.20 | 17.41 | 15.11 | 6.51 | 12.34 | little vitiation of both horns |
| H69⑥ Waterlogged | 24.90 | 19.07 | 10.50 | 7.03 | 6.36 | little vitiation of both horns, big crown |
| H69⑥ Waterlogged | 36.48 | 17.16 | 15.27 | 7.37 | 14.99 | little vitiation of both horns, shrinked crown |
| H69⑥ Waterlogged | 39.37 | 17.11 | 11.45 | 7.28 | 12.76 | crooked |
| H69⑥ Waterlogged | 30.49 | 18.78 | 13.74 | 6.98 | 12.12 | crooked |
| H69⑥ Waterlogged | 34.02 | 19.83 | 10.15 | 7.11 | 14.70 | pressed, crooked |
| H69⑥ Waterlogged | 31.38 | 17.50 | 16.38 | 7.15 | 13.18 | little vitiation of both horns |
| H69⑥ Waterlogged | 27.17 | 16.51 | 13.43 | 6.04 | 10.47 | little vitiation of both horns |
| H69⑥ Waterlogged | 35.07 | 16.41 | 17.14 | 5.87 | 11.80 | little vitiation of both horns |
| H69⑥ Waterlogged | 32.09 | 17.80 | 15.52 | 6.65 | 13.29 | little vitiation of both horns |
| H69⑥ Waterlogged | 31.80 | 18.40 | 17.78 | 6.89 | 14.16 | little vitiation of both horns |
| H69⑥ Waterlogged | 33.56 | 17.08 | 14.49 | 6.63 | 12.64 | little vitiation of both horns |
| H69⑥ Waterlogged | 31.94 | 19.28 | 13.23 | 8.99 | 7.24 | little vitiation of both horns |
| H69⑥ Waterlogged | 30.87 | 15.74 | 16.05 | 6.50 | 11.88 | little vitiation of both horns |
| H69⑥ Waterlogged | 33.77 | 15.70 | 13.70 | 6.80 | 10.87 | crooked |
| H69⑥ Waterlogged | 34.11 | 16.80 | 15.86 | 6.67 | 13.40 |  |
| H69⑥ Waterlogged | 34.14 | 18.15 | 15.80 | 7.01 | 13.30 | little vitiation of both horns |
| H69⑥ Waterlogged | 34.77 | 17.29 | 17.32 | 5.67 | 11.63 | little vitiation of both horns |
| H69⑥ Waterlogged | 33.71 | 16.02 | 10.04 | 6.96 | 13.09 | pressed, crooked |
| H69⑥ Waterlogged | 33.80 | 16.77 | 12.81 | 5.59 | 14.24 | little vitiation of both horns |
| H69⑥ Waterlogged | 33.05 | 17.30 | 14.86 | 5.94 | 13.92 | little vitiation of both horns |
| H69⑥ Waterlogged | 33.62 | 13.51 | 16.88 | 6.32 | 11.37 | crooked |
| H69⑥ Waterlogged | 37.93 | 19.23 | 12.69 | 6.16 | 12.63 | little vitiation of both horns |
| H69⑥ Waterlogged | 24.80 | 16.14 | 10.06 | 6.61 | 8.21 | little vitiation of both horns |
| H69⑥ Waterlogged | 34.82 | 17.65 | 18.30 | 6.30 | 15.10 |  |
| H69⑥ Waterlogged | 33.79 | 16.07 | 14.51 | 6.54 | 12.97 | little vitiation of both horns |
| H69⑥ Waterlogged | 32.22 | 17.98 | 14.29 | 5.70 | 11.16 | crooked |
| H69⑥ Waterlogged | 34.46 | 16.01 | 14.86 | 6.13 | 12.28 | little vitiation of both horns |
| H69⑥ Waterlogged | 24.27 | 15.14 | 8.60 | 6.15 | 7.37 | little vitiation of both horns |
| H69⑥ Waterlogged | 37.58 | 14.97 | 18.31 | 6.19 | 11.73 | little vitiation of both horns, crooked |
| H69⑥ Waterlogged | 34.79 | 16.68 | 14.57 | 6.62 | 14.44 | little vitiation of both horns |
| H69⑥ Waterlogged | 32.92 | 12.58 | 9.28 | 6.10 | 11.10 | little vitiation of both horns, crooked, pressed |
| H69⑥ Waterlogged | 32.82 | 17.66 | 15.45 | 5.82 | 10.62 | little vitiation of both horns, crooked |
| H69⑥ Waterlogged | 31.77 | 15.13 | 15.82 | 5.83 | 9.68 | crooked |
| H69⑥ Waterlogged | 31.19 | 20.71 | 13.50 | 8.30 | 9.42 | little vitiation of both horns |
| H69⑥ Waterlogged | 30.68 | 17.70 | 12.85 | 8.27 | 8.16 | little vitiation of both horns |
| H69⑥ Waterlogged | 29.17 | 18.20 | 7.56 | 5.52 | 10.92 | little vitiation of both horns, pressed |
| H69⑥ Waterlogged | 30.85 | 16.99 | 14.98 | 5.71 | 10.95 |  |
| H69⑥ Waterlogged | 27.60 | 17.10 | 9.40 | 8.16 | 6.21 | little vitiation of both horns, big crown |
| H69⑥ Waterlogged | 31.71 | 18.78 | 8.73 | 5.72 | 8.41 | vitiation of the base |
| H69⑥ Waterlogged | 29.59 | 13.94 | 9.03 | 7.53 | 5.88 |  |
| H69⑥ Waterlogged | 36.63 | 18.54 | 14.30 | 6.37 | 12.45 |  |
| H69⑥ Waterlogged | 34.88 | 16.58 | 15.19 | 7.40 | 12.27 | crooked |
| H69⑥ Waterlogged | 32.64 | 15.67 | 14.72 | 6.23 | 13.52 | little vitiation of both horns |
| H69⑥ Waterlogged | 31.01 | 16.70 | 10.50 | 9.28 | 7.88 |  |
| H69⑥ Waterlogged | 31.73 | 16.87 | 15.65 | 6.11 | 12.92 |  |
| H69⑥ Waterlogged | 33.81 | 19.85 | 9.69 | 6.11 | 12.29 | pressed, big base |
| H69⑥ Waterlogged | 26.98 | 16.18 | 13.00 | 6.10 | 10.22 | little vitiation of both horns |
| H69⑥ Waterlogged | 35.97 | 13.90 | 13.02 | 5.20 | 8.98 | little vitiation of both horns |
| H69⑥ Waterlogged | 31.87 | 16.95 | 10.25 | 6.80 | *Not determined* | little vitiation of both horns and the base, crooked, pressed |
| H69⑥ Waterlogged | 26.81 | 16.20 | 14.48 | 4.67 | *Not determined* | little vitiation of both horns and the base |
| H69⑥ Waterlogged | 38.44 | 17.97 | 13.71 | 5.84 | 13.49 | little vitiation of both horns |
| H69⑥ Waterlogged | 28.93 | 17.45 | 13.85 | 6.50 | 11.12 | little vitiation of both horns |
| H69⑥ Waterlogged | 37.43 | 17.39 | 16.50 | 6.67 | 14.21 | crooked |
| H69⑥ Waterlogged | 32.77 | 17.25 | 16.00 | 6.33 | 13.16 |  |
| H69⑥ Waterlogged | 31.03 | 16.91 | 13.95 | 7.08 | 12.12 | little vitiation of both horns |
| H69⑥ Waterlogged | 22.30 | 12.73 | 12.99 | 5.53 | 9.03 | little vitiation of both horns |
| H69⑥ Waterlogged | 31.09 | 18.78 | 12.93 | 7.54 | 7.24 | little vitiation of both horns |
| H69⑥ Waterlogged | 35.68 | 16.37 | 11.81 | 6.71 | 8.17 |  |
| H69⑥ Waterlogged | 34.43 | 19.22 | 12.28 | 6.85 | 14.31 | little vitiation of both horns |
| H69⑥ Waterlogged | 32.86 | 16.59 | 13.56 | 5.86 | 13.36 |  |
| H69⑥ Waterlogged | 29.53 | 17.40 | 8.65 | 7.58 | 7.31 | little vitiation of both horns, pressed |
| H69⑥ Waterlogged | 36.89 | 17.45 | 9.77 | 9.34 | 8.26 | little vitiation of both horns |
| H69⑥ Waterlogged | 30.95 | 14.73 | 11.83 | 5.84 | 9.82 |  |
| H69⑥ Waterlogged | 35.40 | 16.29 | 13.19 | 5.71 | 12.98 | little vitiation of both horns |
| H69⑥ Waterlogged | 29.35 | 17.68 | 12.35 | 6.64 | 7.66 | little vitiation of both horns |
| H69⑥ Waterlogged | 31.90 | 16.54 | 11.45 | 8.32 | 7.71 | little vitiation of both horns |
| H69⑥ Waterlogged | 38.04 | 13.94 | 17.23 | 5.68 | 11.57 | little vitiation of both horns, crooked |
| H69⑥ Waterlogged | 35.70 | 15.01 | 12.50 | 6.26 | 9.62 | crooked |
| H69⑥ Waterlogged | 32.89 | 16.81 | 15.82 | 6.53 | 12.56 |  |
| H69⑥ Waterlogged | 38.41 | 20.87 | 16.02 | 7.50 | 15.33 | little vitiation of both horns |
| H69⑥ Waterlogged | 22.88 | 15.06 | 9.04 | 4.73 | 5.60 | little vitiation of both horns |
| H69⑥ Waterlogged | 34.29 | 19.63 | 14.88 | 6.02 | 9.33 |  |
| H69⑥ Waterlogged | 38.03 | 15.67 | 19.31 | 7.14 | 12.15 | little vitiation of both horns, crooked |
| H69⑥ Waterlogged | 31.87 | 17.47 | 14.44 | 6.80 | 11.08 | little vitiation of both horns |
| H69⑥ Waterlogged | 30.56 | 14.64 | 12.55 | 6.93 | 10.32 |  |
| H69⑥ Waterlogged | 26.98 | 12.20 | 7.96 | 5.26 | 6.35 | little vitiation of both horns |
| H69⑥ Waterlogged | 24.75 | 13.16 | 10.44 | 3.48 | 5.99 | crooked, carbonized? |
| H69⑥ Waterlogged | 20.55 | 21.10 | 9.38 | 8.59 | 9.52 |  |
| H69⑥ Waterlogged | 25.44 | 12.95 | 8.81 | 5.34 | 8.19 | little vitiation of one horn |
| H69⑥ Waterlogged | 35.51 | 17.96 | 14.03 | 6.48 | 12.25 | little vitiation of both horns |
| H69⑥ Waterlogged | 34.93 | 17.63 | 14.94 | 6.44 | 12.71 | little vitiation of both horns |
| H69⑥ Waterlogged | 29.42 | 15.01 | *Not determined* | 6.14 | 13.02 | little vitiation of both horns |
| H69⑥ Waterlogged | 32.16 | 12.85 | 9.59 | 6.72 | 4.82 | little vitiation of both horns |
| H69⑥ Waterlogged | 35.78 | 17.72 | 9.38 | 7.63 | 7.78 | little vitiation of both horns |
| H69⑥ Waterlogged | 37.39 | 20.96 | 8.24 | 8.36 | 8.71 |  |
| H69⑥ Waterlogged | 32.10 | 15.74 | 16.43 | 5.89 | 12.13 | little vitiation of both horns |
| H69⑥ Waterlogged | 32.56 | 17.52 | 14.90 | 6.30 | 12.70 | little vitiation of both horns |
| H69⑥ Waterlogged | 31.46 | 17.03 | 13.79 | 6.45 | 11.54 | little vitiation of both horns, crooked |
| H69⑥ Waterlogged | 34.31 | 18.25 | 15.16 | 5.44 | 10.21 | little vitiation of both horns |
| H69⑥ Waterlogged | 32.53 | 16.28 | 14.94 | 5.96 | *Not determined* | little vitiation of both horns and the base |
| H69⑥ Waterlogged | 26.75 | 16.44 | 11.98 | 5.82 | 10.94 | little vitiation of both horns |
| H69⑥ Waterlogged | 31.25 | 14.83 | 14.62 | 5.45 | 10.34 | little vitiation of both horns |
| H69⑥ Waterlogged | 34.13 | 19.12 | 12.85 | 6.65 | 14.04 | little vitiation of both horns |
| H69⑥ Waterlogged | 31.25 | 16.54 | 14.76 | 6.56 | 9.42 | little vitiation of both horns |
| H69⑥ Waterlogged | 29.80 | 16.64 | 12.76 | 6.63 | 9.91 | little vitiation of both horns |
| H69⑥ Waterlogged | 37.21 | 17.72 | 15.43 | 6.62 | *Not determined* | little vitiation of both horns and the base, crooked |
| H69⑥ Waterlogged | 35.52 | 15.34 | 15.41 | 6.76 | *Not determined* | little vitiation of both horns and the base |
| H69⑥ Waterlogged | 27.75 | 12.52 | 10 .50 | 4.60 | 6.32 |  |
| H69⑥ Waterlogged | 35.79 | 20.85 | 12.8 3 | 6.22 | 13.90 | little vitiation of both horns |
| H69⑥ Waterlogged | 33.09 | 19.17 | 14.14 | 7.93 | 6.63 | little vitiation of both horns, big crown, small base |
| H69⑥ Waterlogged | 29.83 | 16.72 | 9.27 | 5.94 | 11.16 | pressed, crooked |
| H69⑥ Waterlogged | 28.17 | 17.35 | 11.72 | 6.44 | 4.18 |  |
| H69⑥ Waterlogged | 33.56 | 16.34 | 15.51 | 6.44 | 12.82 | little vitiation of both horns |
| H69⑥ Waterlogged | 27.11 | 14.42 | 11.94 | 7.33 | 5.86 |  |
| H69⑥ Waterlogged | 31.09 | 18.49 | 12.83 | 5.83 | 13.44 | little vitiation of both horns |
| H69⑥ Waterlogged | 33.21 | 18.14 | 15.79 | 6.52 | 13.39 | little vitiation of both horns |
| H69⑥ Waterlogged | 34.05 | 19.77 | 11.32 | 7.12 | 14.11 | little vitiation of both horns |
| H69⑥ Waterlogged | 34.74 | 16.71 | 16.36 | 5.87 | *Not determined* | little vitiation of both horns and the base |
| H69⑥ Waterlogged | 35.23 | 16.96 | 15.37 | 7.85 | 12.44 | little vitiation of both horns |
| H69⑥ Waterlogged | 32.85 | 15.94 | 14.78 | 5.81 | 11.23 | little vitiation of both horns |
| H69⑥ Waterlogged | 34.35 | 19.86 | 12.97 | 7.39 | 12.98 | little vitiation of both horns, crooked |
| H69⑥ Waterlogged | 33.83 | 16.31 | 11.00 | 6.05 | 9.98 | little vitiation of both horns |
| H69⑥ Waterlogged | 30.90 | 13.31 | 17.15 | 6.34 | 10.86 | little vitiation of both horns, crooked |
| H69⑥ Waterlogged | 30.67 | 16.35 | 11.65 | 7.26 | 7.86 | little vitiation of both horns |
| H69⑥ Waterlogged | 29.03 | 17.53 | 14.45 | 6.47 | 11.85 | little vitiation of both horns |
| H69⑥ Waterlogged | 35.60 | 16.85 | 15.03 | 5.27 | 8.07 | little vitiation of both horns and the base |
| H69⑥ Waterlogged | 32.35 | 15.72 | 16.73 | 5.85 | 11.93 | little vitiation of both horns |
| H69⑥ Waterlogged | 27.87 | 13.11 | 13.73 | 5.66 | *Not determined* | little vitiation of both horns and the base |
| H69⑥ Waterlogged | 19.66 | 12.13 | 11.18 | 5.02 | 7.77 | little vitiation of both horns |
| H69⑥ Waterlogged | 35.91 | 19.64 | 10.29 | 6.14 | 14.51 | little vitiation of both horns |
| H69⑥ Waterlogged | 35.76 | 18.54 | 14.51 | 6.44 | 10.86 | little vitiation of both horns |
| H69⑥ Waterlogged | 29.82 | 16.54 | 11.80 | 5.74 | 12.55 | little vitiation of both horns |
| H69⑥ Waterlogged | 38.26 | 16.15 | 16.80 | 6.44 | 12.24 | little vitiation of both horns |
| H69⑥ Waterlogged | 32.50 | 15.95 | 16.83 | 6.45 | 13.88 | little vitiation of both horns |
| H69⑥ Waterlogged | 34.69 | 15.65 | 14.87 | 6.92 | *Not determined* | little vitiation of both horns and the base, crooked |
| H69⑥ Waterlogged | 33.88 | 17.79 | 14.95 | 6.93 | 12.28 | little vitiation of both horns |
| H69⑥ Waterlogged | 20.41 | 14.85 | 8.65 | 6.15 | 6.76 | little vitiation of both horns |
| H69⑥ Waterlogged | 31.24 | 18.98 | 11.61 | 7.28 | 8.11 | little vitiation of both horns |
| H69⑥ Waterlogged | 32.46 | 17.00 | 14.70 | 6.93 | 12.12 | little vitiation of both horns |
| H69⑥ Waterlogged | 34.13 | 17.21 | 14.76 | 6.42 | 13.64 |  |
| H69⑥ Waterlogged | 29.68 | 16.19 | 9.39 | 6.45 | 6.62 | little vitiation of both horns |
| H69⑥ Waterlogged | 31.60 | 17.16 | 13.02 | 6.59 | *Not determined* | vitiation of the base |
| H69⑥ Waterlogged | 32.96 | 20.94 | 9.46 | 7.28 | 14.05 | pressed, crooked |
| H69⑥ Waterlogged | 28.91 | 12.49 | 9.48 | 7.79 | 4.52 | big crown, small base |
| H69⑥ Waterlogged | 27.80 | 16.34 | 11.67 | 6.91 | 8.40 | little vitiation of both horns |
| H69⑥ Waterlogged | 29.15 | 15.63 | *Not determined* | 7.12 | 6.39 |  |
| H69⑥ Waterlogged | 29.77 | 17.54 | 11.85 | 6.43 | 6.77 |  |
| H69⑥ Waterlogged | 32.27 | 14.16 | 6.88 | 5.03 | *Not determined* | no base |
| H69⑥ Waterlogged | 32.62 | 18.23 | 10.31 | 6.53 | 9.38 | little vitiation of both horns |
| H69⑥ Waterlogged | 28.08 | 14.06 | 11.50 | 5.67 | 8.75 |  |
| H69⑥ Waterlogged | 28.93 | 16.82 | 11.58 | 5.93 | *Not determined* | vitiation of the base |
| H69⑥ Waterlogged | 38.78 | 13.77 | *Not determined* | 6.18 | 12.88 |  |
| H69⑥ Waterlogged | 34.01 | 18.90 | 8.86 | 8.37 | 5.06 |  |
| H69⑥ Waterlogged | 34.35 | 14.18 | 18.95 | 6.42 | 14.67 | little vitiation of both horns, crooked |
| H69⑥ Waterlogged | 32.93 | 13.52 | 16.74 | 6.58 | *Not determined* | little vitiation of both horns and the base |
| H69⑥ Waterlogged | 35.90 | 18.90 | 16.25 | 7.59 | 13.02 | little vitiation of both horns |
| H69⑥ Waterlogged | 34.08 | 14.67 | 14.84 | 6.96 | 12.23 | little vitiation of both horns, crooked |
| H69⑥ Waterlogged | 28.17 | 18.68 | 15.41 | 3.04 | 14.31 | little vitiation of both horns |
| H69⑥ Waterlogged | 33.12 | 15.03 | 14.90 | 5.80 | 11.86 | little vitiation of both horns |
| H69⑥ Waterlogged | 24.46 | 16.40 | 9.38 | 6.84 | 9.50 | little vitiation of both horns, big crown and base |
| H69⑥ Waterlogged | 34.93 | 16.92 | 14.89 | 5.43 | 13.56 | little vitiation of both horns |
| H69⑥ Waterlogged | 33.63 | 17.43 | 16.04 | 6.61 | 13.52 | little vitiation of both horns |
| H69⑥ Waterlogged | 34.50 | 18.64 | 14.07 | 6.89 | 14.32 | little vitiation of both horns |
| H69⑥ Waterlogged | 34.91 | 18.46 | 8.82 | 8.24 | 9.01 | little vitiation of both horns, pressed |
| H69⑥ Waterlogged | 34.50 | 18.91 | 16.02 | 7.08 | 14.19 | little vitiation of both horns, crooked |
| H69⑥ Waterlogged | 31.98 | 16.04 | 16.12 | 6.33 | 14.81 | little vitiation of both horns |
| H69⑥ Waterlogged | 30.36 | 17.95 | 9.02 | 7.72 | 7.53 |  |
| H69⑥ Waterlogged | 31.96 | 21.08 | 14.73 | 6.70 | 14.43 | little vitation of both horns |
| H69⑥ Waterlogged | 32.00 | 18.65 | 13.17 | 6.85 | 10.64 | little vitiation of both horns |
| H69⑥ Waterlogged | 31.49 | 16.58 | 14.35 | 6.50 | 12.31 |  |
| H69⑥ Waterlogged | 34.68 | 16.74 | 16.37 | 6.23 | *Not determined* | crooked, no base |
| H69⑥ Waterlogged | 27.60 | 16.61 | 15.63 | 5.83 | 13.29 | little vitiation of both horns |
| H69⑥ Waterlogged | 35.85 | 15.17 | 16.59 | 7.03 | *Not determined* | little vitiation of both horns and the base |
| H69⑥ Waterlogged | 34.22 | 16.38 | 16.74 | 6.41 | 13.73 | little vitiation of both horns |
| H69⑥ Waterlogged | 34.74 | 17.80 | 17.00 | 6.60 | 14.36 | little vitiation of both horns |
| H69⑥ Waterlogged | 29.34 | 14.63 | 12.32 | 3.82 | 8.83 | crooked |
| H69⑥ Waterlogged | 32.93 | 17.18 | 14.71 | 6.95 | 13.45 | little vitiation of both horns |
| H69⑥ Waterlogged | 31.67 | 16.48 | 11.70 | 6.07 | 9.18 | little vitiation of both horns |
| H69⑥ Waterlogged | 34.76 | 18.21 | 12.53 | 5.89 | 12.47 | little vitiation of both horns |
| H69⑥ Waterlogged | 30.70 | 15.18 | 14.76 | 6.88 | 12.88 | little vitiation of both horns |
| H69⑥ Waterlogged | 29.67 | 19.56 | 9.36 | 6.75 | 7.12 | little vitiation of both horns |
| H69⑥ Waterlogged | 35.20 | 15.10 | 16.53 | 6.56 | 13.15 | little vitiation of both horns |
| H69⑥ Waterlogged | 35.60 | 19.53 | 16.86 | 6.32 | 13.03 | little vitiation of both horns |
| H69⑥ Waterlogged | 28.53 | 16.35 | 15.53 | 6.22 | 13.78 | little vitiation of both horns |
| H69⑥ Waterlogged | 30.03 | 16.24 | 16.37 | 5.44 | 11.20 |  |
| H69⑥ Waterlogged | 34.42 | 18.05 | 14.48 | 6.36 | 13.63 |  |
| H69⑥ Waterlogged | 34.70 | 18.60 | 13.39 | 6.88 | 15.31 |  |
| H69⑥ Waterlogged | 37.28 | 18.89 | 15.17 | 4.67 | *Not determined* |  |
| H69⑥ Waterlogged | 33.63 | 13.55 | 15.94 | 5.66 | 12.50 | little vitiation of both horns |
| H69⑥ Waterlogged | 36.72 | 18.21 | 16.97 | 5.55 | 12.85 | little vitiation of both horns |
| H69⑥ Waterlogged | 33.03 | 15.68 | 12.00 | 5.52 | 12.22 |  |
| H69⑥ Waterlogged | 34.44 | 16.74 | 15.52 | 5.98 | 13.37 |  |
| H69⑥ Waterlogged | 29.75 | 16.94 | 12.78 | 6.64 | 12.30 | little vitiation of both horns |
| H69⑥ Waterlogged | 35.03 | 17.46 | 11.23 | 7.07 | *Not determined* | little vitiation of both horns |
| H69⑥ Waterlogged | 31.61 | 17.10 | 14.70 | 6.81 | 12.39 | little vitiation of both horns |
| H69⑥ Waterlogged | 29.76 | 15.24 | 9.35 | 6.82 | 6.09 |  |
| H69⑥ Waterlogged | 31.91 | 15.13 | 15.50 | 5.48 | 14.63 |  |
| H69⑥ Waterlogged | 34.06 | 16.51 | 15.35 | 6.24 | 12.55 |  |
| H69⑥ Waterlogged | 28.28 | 13.01 | 13.44 | 5.62 | 9.52 |  |
| H69⑥ Waterlogged | 37.48 | 16.96 | 17.34 | 5.79 | 13.22 | little vitiation of both horns, crooked |
| H69⑥ Waterlogged | 34.93 | 16.38 | 17.78 | 6.81 | 13.30 | little vitiation of both horns, crooked |
| H69⑥ Waterlogged | 30.34 | 17.62 | 15.06 | 6.00 | 13.15 | little vitiation of both horns |
| H69⑥ Waterlogged | 29.20 | 15.60 | 14.29 | 6.82 | 12.78 | vitiation of one horn and little vitiation of the other one |
| H69⑥ Waterlogged | 30.62 | 18.08 | 15.68 | 6.43 | 13.06 | little vitiation of one horn |
| H69⑥ Waterlogged | 33.08 | 15.69 | 17.13 | 6.18 | 13.04 | little vitiation of both horns |
| H69⑥ Waterlogged | 34.51 | 17.09 | 10.84 | 7.04 | 8.86 | little vitiation of both horns |
| H69⑥ Waterlogged | 25.02 | 12.84 | 12.27 | 6.04 | 7.34 | little vitiation of both horns, crooked |
| H69⑥ Waterlogged | 36.26 | 17.40 | 10.33 | 9.18 | 8.81 | little vitiation of both horns, big crown |
| H69⑥ Waterlogged | 29.23 | 12.44 | 9.21 | 6.62 | 6.12 |  |
| H69⑥ Waterlogged | 32.97 | 16.41 | 15.85 | 6.94 | 13.10 | little vitiation of both horns |
| H69⑥ Waterlogged | 34.49 | 17.63 | 15.82 | 6.56 | 12.42 | little vitiation of both horns |
| H69⑥ Waterlogged | 23.54 | 19.60 | 11.41 | 6.96 | 6.08 | little vitiation of both horns |
| H69⑥ Waterlogged | 27.72 | 16.20 | 16.32 | 6.13 | 12.87 | little vitiation of both horns |
| H69⑥ Waterlogged | 29.10 | 16.49 | 9.01 | 6.70 | 6.99 | little vitiation of both horns |
| H69⑥ Waterlogged | 29.94 | 19.42 | 8.42 | 7.61 | 8.26 | little vitiation of both horns, pressed |
| H69⑥ Waterlogged | 33.70 | 18.15 | 17.61 | 7.07 | 14.16 | little vitiation of both horns |
| H69⑥ Waterlogged | 27.04 | 14.39 | 13.09 | 6.15 | 10.86 | little vitiation of both horns |
| H69⑥ Waterlogged | 29.39 | 17.55 | 8.94 | 8.91 | 8.07 | little vitiation of both horns |
| H69⑥ Waterlogged | 30.68 | 16.36 | 11.47 | 7.56 | 11.46 | little vitiation of both horns |
| H69⑥ Waterlogged | 29.15 | 15.78 | 14.63 | 4.70 | 9.59 | crooked |
| **Mean** | 32.20 | 16.84 | 13.60 | 6.48 | 11.16 |  |  |
| **±SD** | 3.45 | 1.92 | 2.70 | 0.91 | 2.58 |  |  |
| **Number** | 261 | 261 | 256 | 261 | 245 |  |  |
| **Sample** | **Length (mm)** | **Height (mm)** | **Thickness (mm)** | **Top Diameter (mm)** | **Bottom Diameter (mm)** | **Comments** | **Type** |
| H69⑥ Preserved | 37.17 | 21.76 | 12.37 | 7.02 | *Not determined* |  | *Trapa bispinosa* Roxb., collected from the Tianluoshan site |
| H69⑥ Preserved | 34.02 | 18.50 | 13.89 | 6.67 | 14.49 |  |
| H69⑥ Preserved | 34.04 | 19.68 | 15.85 | 7.01 | 13.85 | little vitiation of one horn |
| H69⑥ Preserved | 33.30 | 18.47 | 13.84 | 7.46 | 13.59 |  |
| H69⑥ Preserved | 34.23 | 18.42 | 12.03 | 6.34 | 9.35 |  |
| H69⑥ Preserved | 31.88 | 20.05 | 7.90 | 7.14 | 7.56 | little vitiation of one horn |
| H69⑥ Preserved | 35.59 | 20.63 | 19.48 | 7.08 | 15.03 |  |
| H69⑥ Preserved | 31.54 | 20.78 | 12.78 | 6.94 | 14.87 | little vitiation of one horn |
| H69⑥ Preserved | 34.10 | 18.35 | 11.71 | 8.27 | 10.31 |  |
| H69⑥ Preserved | 37.07 | 21.27 | 16.97 | 7.71 | 14.82 |  |
| H69⑥ Preserved | 36.90 | 19.99 | 18.68 | 9.53 | 15.97 | little vitiation of one horn |
| H69⑥ Preserved | 23.70 | 19.70 | 13.76 | 4.81 | 11.02 | little vitiation of one horn |
| H69⑥ Preserved | 33.44 | 21.79 | 9.28 | 6.56 | 12.41 |  |
| H69⑥ Preserved | 32.87 | 17.74 | 15.34 | 7.02 | 13.07 |  |
| H69⑥ Preserved | 35.50 | 16.97 | 15.10 | 6.44 | 12.38 |  |
| H69⑥ Preserved | 35.84 | 16.89 | 16.29 | 7.69 | 14.67 |  |
| H69⑥ Preserved | 35.69 | 13.64 | 15.68 | 7.00 | 13.07 |  |
| H69⑥ Preserved | 38.88 | 19.27 | 17.48 | 8.34 | 15.91 |  |
| H69⑥ Preserved | 33.30 | 18.46 | 15.45 | 6.45 | 13.18 |  |
| H69⑥ Preserved | 29.67 | 16.93 | 12.59 | 3.67 | 11.49 |  |
| H69⑥ Preserved | 34.93 | 18.23 | 15.36 | 7.42 | 11.68 |  |
| H69⑥ Preserved | 35.43 | 16.94 | 14.83 | 5.87 | 13.49 |  |
| H69⑥ Preserved | 35.83 | 17.43 | 16.78 | 6.63 | 15.43 |  |
| H69⑥ Preserved | 34.86 | 19.83 | 11.12 | 9.34 | 9.55 |  |
| H69⑥ Preserved | 33.91 | 16.84 | 16.19 | 6.85 | 12.48 |  |
| H69⑥ Preserved | 34.06 | 12.81 | 16.25 | 6.85 | 9.43 |  |
| H69⑥ Preserved | 34.35 | 19.22 | 15.68 | 6.47 | 15.83 |  |
| H69⑥ Preserved | 38.08 | 18.80 | 9.01 | 9.54 | 14.18 |  |
| H69⑥ Preserved | 29.45 | 17.65 | 11.00 | 6.75 | 12.47 |  |
| H69⑥ Preserved | 31.95 | 16.93 | 11.98 | 7.05 | 11.08 |  |
| H69⑥ Preserved | 28.40 | 14.48 | 12.20 | 6.94 | 8.92 |  |
| H69⑥ Preserved | 39.21 | 21.53 | 11.94 | 7.77 | 15.52 |  |
| H69⑥ Preserved | 30.72 | 15.91 | 14.78 | 6.08 | 11.49 | little vitiation of both horns |
| H69⑥ Preserved | 34.96 | 18.30 | 10.20 | 6.81 | 12.47 |  |
| H69⑥ Preserved | 33.88 | 17.18 | 18.13 | 8.17 | 12.29 |  |
| H69⑥ Preserved | 30.36 | 14.33 | 13.99 | 3.03 | 11.56 |  |
| H69⑥ Preserved | 31.28 | 15.47 | 15.47 | 5.57 | 11.18 |  |
| H69⑥ Preserved | 34.28 | 16.33 | 14.95 | 6.98 | 9.82 |  |
| H69⑥ Preserved | 36.80 | 18.69 | 10.94 | 7.21 | 11.35 |  |
| H69⑥ Preserved | *Not determined* | 16.40 | 13.27 | 4.70 | 13.59 |  |
| H69⑥ Preserved | 32.68 | 16.95 | 14.35 | 6.66 | 11.90 |  |
| H69⑥ Preserved | 32.94 | 16.94 | 16.42 | 6.83 | 13.21 |  |
| H69⑥ Preserved | 35.37 | 19.03 | 14.80 | 6.33 | 11.54 |  |
| H69⑥ Preserved | 33.49 | 15.39 | *Not determined* | 6.27 | *Not determined* |  |
| H69⑥ Preserved | 24.26 | 17.73 | *Not determined* | 6.50 | 5.67 |  |
| H69⑥ Preserved | 28.69 | 16.15 | 10.31 | 7.56 | 7.20 |  |
| H69⑥ Preserved | 24.86 | 14.92 | 9.41 | 5.97 | 6.37 |  |
| H69⑥ Preserved | 27.27 | 16.30 | 10.51 | 6.04 | 12.26 | little vitiation of one horn |
| H69⑥ Preserved | 28.14 | 15.76 | 12.41 | 7.07 | 10.94 | little vitiation of one horn |
| H69⑥ Preserved | 29.06 | 12.13 | 16.61 | 5.21 | 9.89 |  |
| H69⑥ Preserved | 25.96 | 15.99 | 10.14 | 7.73 | 6.88 |  |
| H69⑥ Preserved | 27.29 | 12.87 | 11.65 | 6.44 | 6.28 |  |
| H69⑥ Preserved | 26.44 | 13.61 | *Not determined* | 7.97 | 5.32 |  |
| **Mean** | **32.65** | **17.48** | **13.74** | **6.83** | **11.73** |  |  |
| **±SD** | **3.80** | **2.34** | **2.73** | **1.20** | **2.80** |  |  |
| **Number** | **52** | **53** | **50** | **53** | **51** |  |  |
| **Layer 6 summary information for the water chestnuts from Tianluoshan** | | | | | | | |
| **Layer 6 Mean** | **32.28** | **16.95** | **13.62** | **6.54** | **11.26** |  |  |
| **Layer 6 ±SD** | **3.51** | **2.01** | **2.70** | **0.98** | **2.62** |  |  |
| **Layer 6 Number** | **313** | **314** | **306** | **314** | **296** |  |  |
